# ChIPr: accurate prediction of cohesin-mediated 3D genome organization from 2D chromatin features

**DOI:** 10.1186/s13059-023-03158-7

**Published:** 2024-01-12

**Authors:** Ahmed Abbas, Khyati Chandratre, Yunpeng Gao, Jiapei Yuan, Michael Q. Zhang, Ram S. Mani

**Affiliations:** 1 Department of Pathology, UT Southwestern Medical Center, Dallas, TX 75390 USA; 2 Department of Biological Sciences, Center for Systems Biology, The University of Texas at Dallas, Richardson, TX 75080 USA; 3 State Key Laboratory of Experimental Hematology, National Clinical Research Center for Blood Diseases, Haihe Laboratory of Cell Ecosystem, Institute of Hematology and Blood Diseases Hospital, Chinese Academy of Medical Sciences and Peking Union Medical College, Tianjin, 300020 China; 4 Department of Urology, UT Southwestern Medical Center, Dallas, TX 75390 USA; 5 Harold C. Simmons Comprehensive Cancer Center, UT Southwestern Medical Center, Dallas, TX 75390 USA

## Abstract

The three-dimensional genome organization influences diverse nuclear processes. Here we present Chromatin Interaction Predictor (ChIPr), a suite of regression models based on deep neural networks, random forest, and gradient boosting to predict cohesin-mediated chromatin interaction strength between any two loci in the genome. The predictions of ChIPr correlate well with ChIA-PET data in four cell lines. The standard ChIPr model requires three experimental inputs: ChIP-Seq signals for RAD21, H3K27ac, and H3K27me3 but works well with just RAD21 signal. Integrative analysis reveals novel insights into the role of CTCF motif, its orientation, and CTCF binding on cohesin-mediated chromatin interactions.

**Supplementary Information:**

The online version contains supplementary material available at 10.1186/s13059-023-03158-7.

## Background

The three-dimensional (3D) genome organization directly impacts diverse nuclear processes such as transcription, DNA repair, and replication. Therefore, it is crucial to understand how the distal regulatory elements (in the linear genome) interact in 3D space. Several sequencing-based and imaging-based experimental methods have been developed in the last two decades to study the 3D genome organization [1]. Many of the sequencing-based approaches are derived from the chromosome conformation capture (3C) concept [2]. High-throughput chromosome conformation capture (Hi-C) [3] and chromatin interaction analysis by paired-end tag sequencing (ChIA-PET) [4] are some of the commonly used methods to study 3D genome organization. Hi-C detects all possible genome-wide pairwise interactions between loci. By using Hi-C maps, it was observed that chromosomes are partitioned into two compartments, A and B, representing active and inactive chromatin regions, respectively [3]. Analysis of relatively high-resolution Hi-C maps (~40 kbp) resulted in the discovery of self-interacting genomic regions called topologically associating domains (TADs) [5–8]. Much higher resolution Hi-C maps (in the range of 1–5 kbp) have revealed enhancer–promoter contacts [9]. Derivatives of Hi-C, such as Micro-C, provide higher resolution contact maps [10].

Hi-C identifies all chromatin contacts but does not specify the proteins associated with 3D interactions. This is partially addressed by including a chromatin immunoprecipitation (ChIP) step with the Hi-C protocol. For example, ChIA-PET captures genome-wide interactions associated with specific proteins. ChIA-PET has facilitated the discovery of chromatin interactions associated with transcription factors (ER, AR), RNA Polymerase II, and structural proteins such as the cohesin component RAD21 and CTCF [11–14]. However, Hi-C and ChIA-PET experiments are labor-intensive, time-consuming, and expensive [9, 15]. Furthermore, there always exists a possibility that the experiment outcome may not be of the desired quality. The ENCODE portal has provided RAD21 ChIA-PET datasets for about 24 cell lines [12]. However, we still do not have the RAD21 ChIA-PET for many other cell lines. We still do not fully understand the key determinants of cohesin-mediated chromatin interactions. Therefore, we sought to develop a machine learning method to predict cohesin-associated chromatin interactions using simple 2D chromatin and other associated genomic features.

Machine learning has been applied to solve long-standing questions in biology. Notably, the AlphaFold system has been applied to accurately predict the 3D shape of a protein from its amino acid sequence [16]. Several machine learning systems have been developed to understand 3D genome organization (see review articles [17, 18]). For instance, transcription factor and histone modification ChIP-Seq data were used to predict the chromatin interactions between loop-associated ERα binding sites (laERBSs) [19]. Higher-order chromatin organization A/B compartments, originally calculated using Hi-C data [3], have been predicted from epigenetic data, such as DNA methylation microarray, DNase hypersensitivity sequencing, single-cell ATAC sequencing, and single-cell whole-genome bisulfite sequencing [20]. In [21], the authors developed a neural network to predict chromatin structural types (i.e., to which subcompartments [9] the chromatin loci belong) from ChIP-Seq signals. They used the available ENCODE ChIP-seq data for the GM12878 cell line (84 protein binding and 11 histone modification experiments). They have also trained a reduced model using only the 11 histone modification experiments [21]. Moreover, Gradient Boosting regressor was used to predict the interaction frequency between loci of 25 kbp size (the model was shown to work also at 5 kbp resolution) [22]. In the final model, RNA-seq data, CTCF binding, and genomic distance were used as the regression model predictors [22]. In Chromatin Interaction Neural Network (ChINN), DNA sequences of interacting loci were used to predict CTCF-, RNA polymerase II- and Hi-C-associated chromatin interactions [23]. However, to our knowledge, none of the existing computational methods predicts the strength of RAD21-mediated chromatin interactions despite the importance of this cohesin subunit in shaping the 3D genome [24]. Our study fills-in this knowledge gap in the field.

In this study, we present Chromatin Interaction Predictor (ChIPr), a suite of regression models based on deep neural networks (DNN-ChIPr), random forest (RF-ChIPr), and gradient boosting (GB-ChIPr), respectively, to predict the strength of chromatin interactions between any two anchor peaks. Our main assumption is that the interaction strength between any pair of peaks depends on a set of factors that can be easily measured or widely (publicly) available. We hypothesized that the interaction strength between two peaks depends on (A) the enrichment of the protein of interest in the two peaks (feature 1), which can be measured by ChIP-Seq, (B) the enrichment of active and inactive histone modifications (features 2 and 3), which can also be measured by ChIP-Seq, and (C) additional factors that can be easily calculated without any new experimental data, like the genomic distance between the two peaks, the GC content of the two peaks, and the CTCF motif orientation in the two peaks (features 4 to 6). These six features were selected as inputs for our model. The output of ChIPr is the predicted strength of the interaction between any two peaks/regions of interest.

We demonstrate that the predictions of ChIPr correlate well with the original ChIA-PET (as our positive control) interactions at the peak-level resolution and bin sizes of 25 and 5 kbp. We show that ChIPr accurately predicts most of the cell-type-dependent loops identified by either ChIA-PET or Hi-C. Moreover, we have analyzed the importance of each of the model inputs for the model’s prediction accuracy and performed a detailed analysis for the role of CTCF motif orientation and CTCF occupancy in the prevalence and strength of cohesin-mediated chromatin interactions. We report a distinct class of cohesin-mediated chromatin interactions that lack CTCF binding. These loops are significantly enriched for enhancer-enhancer interactions. Our benchmarking studies indicated that ChIPr outperforms other comparable 3D chromatin interaction prediction methods, such as C.Origami [25], Orca [26], and 3Dpredictor [22]. Remarkably, our results demonstrate that, with a single experimental data (RAD21 ChIP-Seq), ChIPr can predict cohesin-mediated chromatin interactions with high accuracy. In addition to cohesin loops (RAD21 ChIA-PET), ChIPr can also accurately predict the results of Hi-C and Micro-C experiments.

## Results

### ChIPr predictions correlate well with the original data at the peak-level resolution

**Fig. 1:**
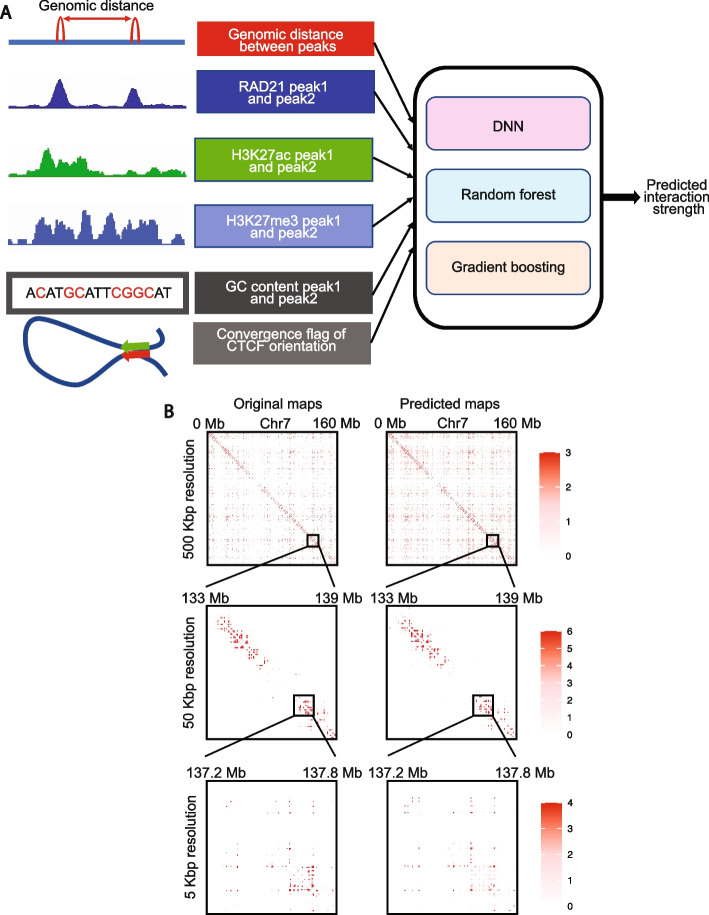
Overview of ChIPr, a regression model with three variants to predict interaction strength between two peaks. **A** A schematic representation of ChIPr showing all the input features, the three regression variants, and the expected output from ChIPr. **B** An example of contact maps constructed from the original ChIA-PET data and the corresponding ones constructed from the predictions of DNN-ChIPr at resolutions of 500, 50, and 5 kbp. Heatmaps in **B** were plotted using HiTC

**Fig. 2:**
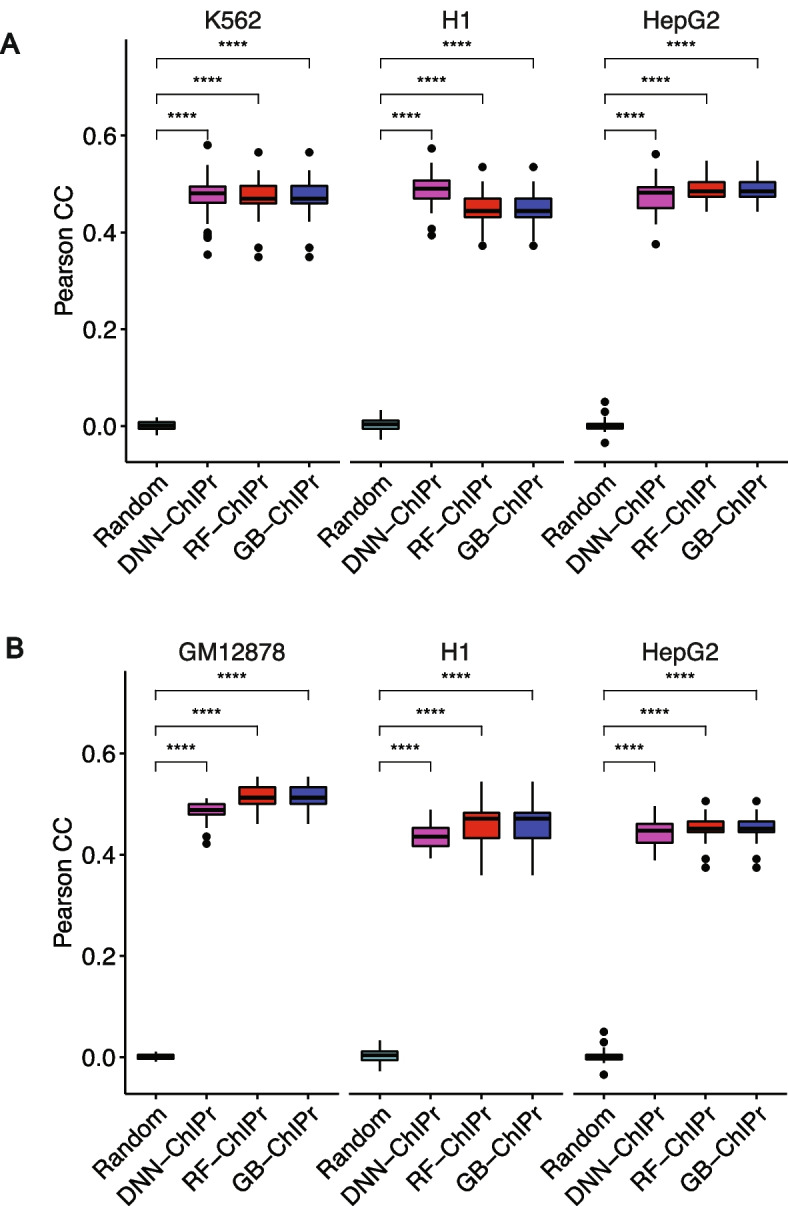
Predicted interactions correlate well with the original ones at the peak-level-resolution. **A** Predicted interactions using the three variants of ChIPr for the cell lines K562, H1, and HepG2 correlate significantly better than the random interactions with the original ChIA-PET interactions of these three cell lines. The predictions in **A** are obtained using models trained on the GM12878 cell line data. **B** Predicted interactions using the three variants of ChIPr for the cell lines GM12878, H1, and HepG2 correlate significantly better than the random interactions with the original ChIA-PET interactions of these three cell lines. The predictions in **B** are obtained using the models trained on the K562 cell line data. ****: *p*-value < 0.0001, Wilcoxon rank sum test

The schematic of the method and a few examples of the contact maps (plotted by HiTC [27]) that can be constructed using the predicted outputs at different resolutions are shown in Fig. 1A and B, respectively. Additional details about the input features and the regression models can be found in the “Methods” section. For each of the three variants of ChIPr—DNN-ChIPr, RF-ChIPr, and GB-ChIPr—we trained two main models using the data of the two cell lines, GM12878 and K562, respectively. We chose GM12878 and K562 because they are two of the best-characterized cell lines in the ENCODE portal [28, 29], with the highest data quality. In addition, using models trained on two different cell lines reduces the inherent biases which might be observed due to the presence of structural variations and mutations in the genome. We used the models trained on the RAD21 ChIA-PET data from GM12878 to predict RAD21 interactions’ strengths in the cell lines K562, H1, and HepG2 using the six inputs described in Fig. 1A—RAD21 ChIP-Seq, H3K27ac ChIP-Seq, H3K27me3 ChIP-Seq, the genomic distance between peaks, GC content, and CTCF motif orientation flag. The CTCF motif orientation flag is an input that is set to “1” if CTCF motif orientations in the two interacting peaks are convergent, and is set to “0” otherwise. Reciprocally, we used the models trained on the RAD21 ChIA-PET data from K562 to predict the strengths of RAD21 interactions in the cell lines GM12878, H1, and HepG2. The RAD21 ChIP-Seq data used in our studies were not derived from RAD21 ChIA-PET data and therefore represent bonafide independent datasets. We have previously shown that ChIA-PET interaction strengths follow a negative binomial distribution [14]. Hence, to evaluate the performance of ChIPr, we generated random values for the interactions’ strengths drawn from negative binomial distributions with the same mean and variance as that of the corresponding original ChIA-PET sample. We measured the correlation coefficient values between the predictions we obtained for the four cell lines (using the models trained on GM12878 and K562 data, respectively) and the original ChIA-PET data. We found that the predicted outputs of the three different variants of ChIPr correlated significantly better with the original data than the randomly generated interactions’ strengths (Fig. 2A, B, Additional file 1: Fig. S1A and B). We also found that the three different regression models—DNN-ChIPr, RF-ChIPr, and GB-ChIPr—yielded comparable results (Fig. 2A, B, Additional file 1: Fig. S1A and B). In addition, the results for the cell lines H1 and HepG2 are quite similar for the models trained on GM12878 and K562 data, respectively (Fig. 2A, B, Additional file 1: Fig. S1A and B). These results showcase the accuracy, reproducibility, and generalizability of ChIPr.

### ChIPr predictions correlate well with the original ChIA-PET data at 25- and 5-kbp bin resolution

Although our goal is to predict the chromatin interactions’ strengths at the peak-level resolution, we can still capture much information at lower resolution. For instance, we can predict TADs using contact maps of 25 and 5 kbp resolutions [9]. Thus, we sought to measure how well the ChIPr outputs correlate with the original data at these lower resolutions.

In [30], HiCRep was developed to assess the reproducibility of Hi-C data taking into account its unique spatial features, such as domain structure and distance dependence. HiCRep minimizes the effect of noise by smoothing the Hi-C maps. It also addresses the impact of distance dependence by dividing the contact maps into strata. It calculates the Pearson correlation coefficient between every two corresponding strata in the two maps being compared. The weighted sum of these Pearson correlation coefficients is called the stratum-adjusted correlation coefficient (SCC). SCC has the same range and interpretation as standard correlation coefficients [30]. In [31], a faster and more computationally efficient version of HiCRep was developed.

**Fig. 3:**
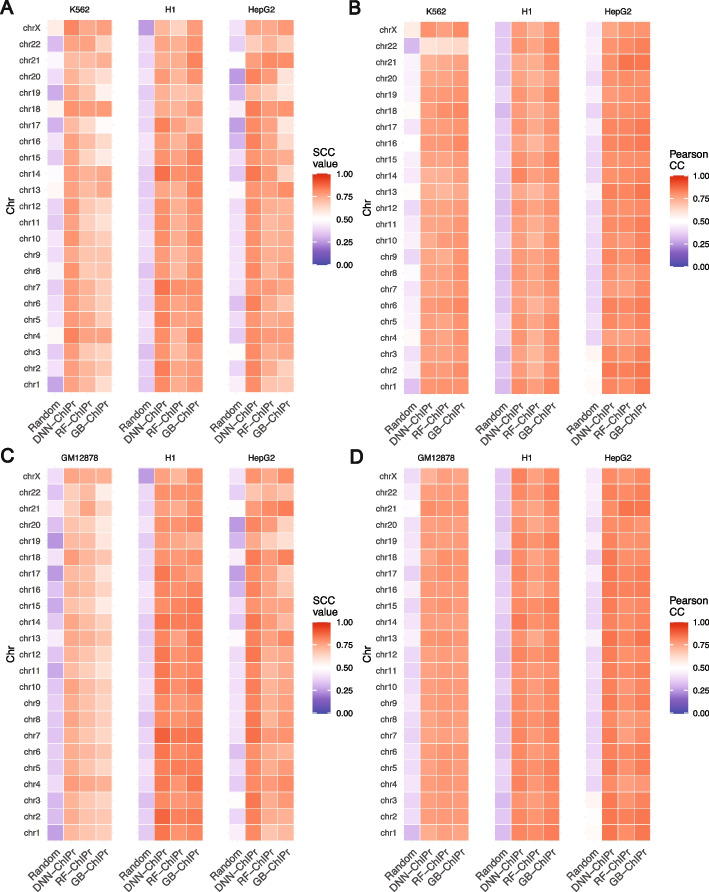
Predicted interactions correlate well with the original ones at the 25 kbp bin resolution. **A**, **B** Comparison between the correlation coefficient values between the original interactions and the predicted ones using the three variants of ChIPr vs. those between the original and randomly generated ones for the three cell lines K562, H1, and HepG2. The correlation coefficients were calculated using stratum-adjusted correlation coefficients (**A**) and Pearson correlation coefficients (**B**), respectively. The predictions in **A** and **B** were generated using the models trained on GM12878 data. **C**, **D** Comparison between the correlation coefficient values between the original interactions and the predicted ones using the three variants of ChIPr vs. those between the original and randomly generated ones for the three cell lines GM12878, H1, and HepG2. The correlation coefficients were calculated using stratum-adjusted correlation coefficients (**C**) and Pearson correlation coefficients (**D**), respectively. The predictions in **C** and **D** were generated using the models trained on K562 data

We used SCC and Pearson correlation coefficients to evaluate the similarity between the original data and the outputs of ChIPr. More specifically, we created interaction maps for the original, predicted, and randomly generated interactions at 25- and 5-kbp bin sizes (see “Methods” for details about constructing contact maps). We measured SCC and Pearson correlation between the original maps vs. the predicted and random ones. For SCC, we set the smoothing window half-size *h* to “2” and the maximum genomic distance to include in calculations to 25 Mbp. We found predicted maps correlate significantly with the original maps than the random ones (Fig. 3A–D, Additional file 1: Fig. S2A and B). All these results show the agreement between original and predicted contact maps. This agreement highlights the ability of ChIPr to reproduce reasonably accurate contact maps with relatively small bin sizes like 25 and 5 kbps.

### ChIPr captures ChIA-PET identified cell-type-dependent interactions

In [12], ChIA-PET was used to study the cohesin-mediated chromatin loops in 24 cell lines. The authors pooled ~125,000 interactions across all the cell lines and found that ~28% of that pan-cell line loop set are variable loops (i.e., cell-type-dependent loops). These variable loops are strong in certain cell types and weaker or near noise level in other types.

**Fig. 4:**
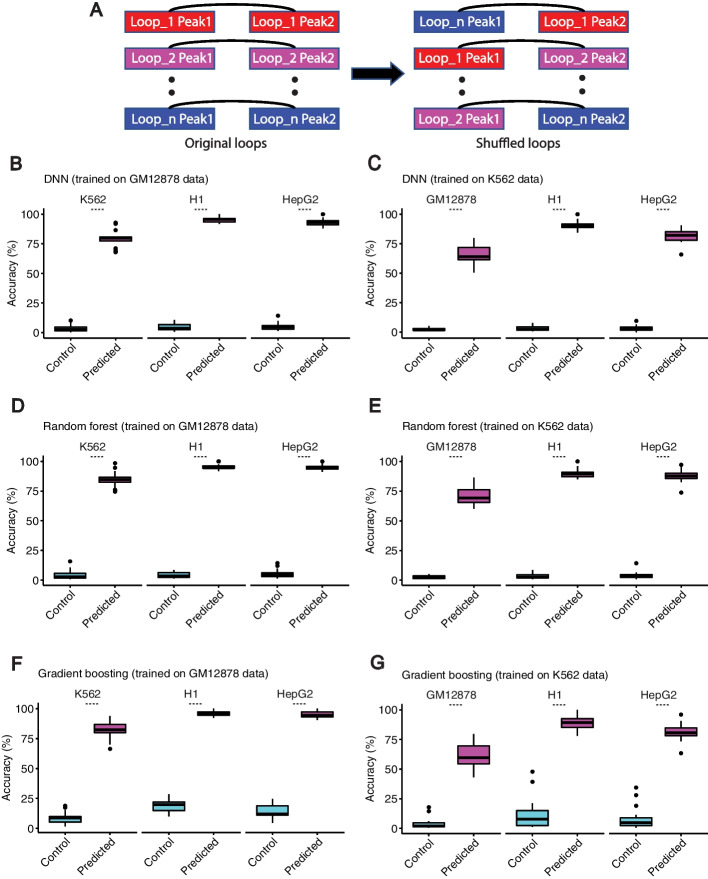
Predicted interactions capture the majority of cell-type-dependent loops. **A** An illustration of how the random control loops were generated for the comparison. **B**–**G** Predicted interactions using DNN-ChIPr (**B** and **C**), RF-ChIPr (**D** and **E**), and GB-ChIPr (**F** and **G**) captured a significantly higher portion of the ChIA-PET identified cell-type-dependent loops vs. randomly introduced loops of the same number for the cell lines K562, H1, and HepG2. The models in panels **B**, **D**, and **F** were trained using the data of GM12878 cell line and the models in panels **C**, **E**, and **G** were trained using the data of K562 cell line. ****: *p*-value < 0.0001, Wilcoxon rank sum test

We investigated the whole list of cell-type-dependent loops to see if they are captured by ChIPr as strong interactions in the corresponding cell types (i.e., interaction strength (PETs) greater than or equal to “3”). As a negative control, we introduced an equal number of random interactions by shuffling the coordinates of the first peak of the cell-type-dependent loops of each chromosome (see Fig. 4A). These randomly introduced loops were not expected to be predicted by ChIPr as strong interactions. We found that, on average, 74, 78.5, and 72.15% of the cell-type-dependent loops are captured in the four cell lines using DNN-ChIPr, RF-ChIPr, and GB-ChIPr, respectively (Fig. 4B–G). On the other hand, 2.3, 2.6, and 4.7% of the randomly introduced interactions were predicted as strong interactions using DNN-ChIPr, RF-ChIPr, and GB-ChIPr, respectively (Fig. 4B–G). These results highlight the utility of ChIPr in predicting cell-type-dependent cohesin-mediated chromatin interactions.

### ChIPr captures both cell-type-dependent and universal cohesin-mediated chromatin interactions

**Fig. 5:**
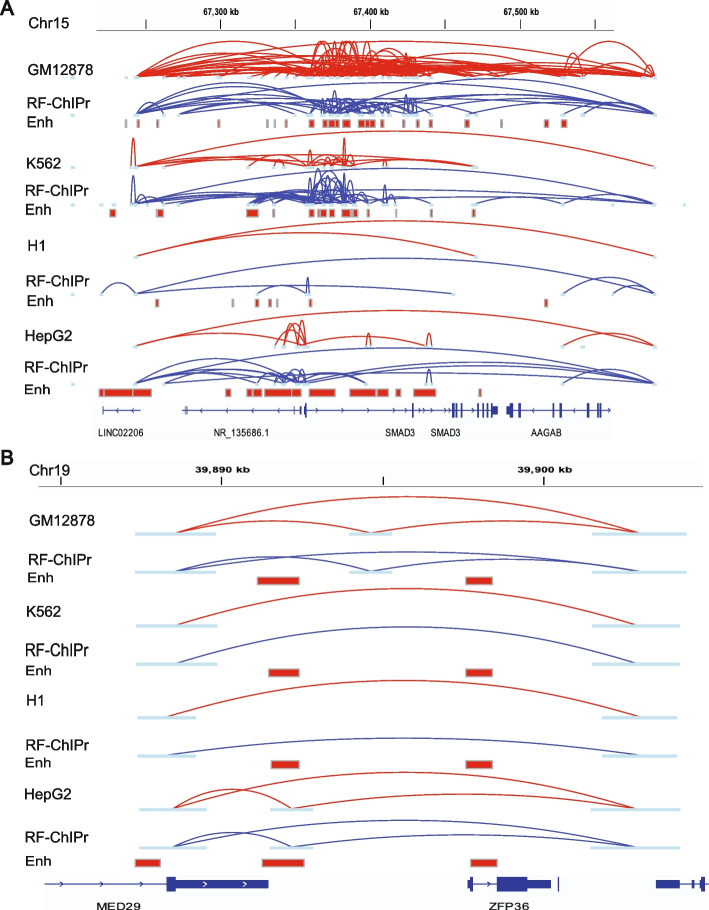
Examples for the predictions of RF-ChIPr for variable and non-variable loops. **A** Predictions of RF-ChIPr are highly similar to the original data for the selected region surrounding *SMAD3* gene. **B** Predictions of RF-ChIPr are highly similar to the original data for the non-variable loops in the region covering the two genes *MED29* and *ZFP36* in the four cell lines GM12878, K562, H1, and HepG2. Interactions shown in **A** and **B** are those having strength ≥ “3”. Red: original loops from RAD21 ChIA-PET data; blue: predicted loops by RF-ChIPr

We further investigated a region around the *SMAD3* gene in the four cell lines GM12878, K562, H1, and HepG2. *SMAD3* functions as a signal transducer in the transforming growth factor-beta (TGF-β) signalling pathway. It also transmits signals from the cell surface to the nucleus to regulate cell proliferation and gene activity [32, 33]. To visually evaluate and show the accuracy of the interaction strength predicted using ChIPr regression model, we compared the interactions from original ChIA-PET data to those predicted by RF-ChIPr model which was trained on GM12878 data (for K562, H1, and HepG2 cell lines) and K562 data (for GM12878 cell line), in *SMAD3* gene region. We found relatively dense, strongly predicted interactions for the cell lines GM12878, K562, and HepG2, which was consistent with the elevated activity of the enhancer elements in the corresponding region in these cell lines (Fig. 5A). On the other hand, we found few interactions in the case of H1, which was also consistent with the reduced activity of the enhancers in the region (Fig. 5A). Similarly, we examined another region covering the two genes *MED29* and *ZFP39*. *MED29* gene encodes for a protein which is a part of the mediator complex and functions in the regulation of transcription of nearly all RNA Polymerase II-dependent genes [32, 33]. On the other hand, *ZFP36* gene encodes for an RNA-binding protein involved in mRNA metabolism pathways [32, 33]. This region comprising a non-variable loop predicted strongly in all of the four cell lines was also in line with the original data (Fig. 5B).

**Fig. 6:**
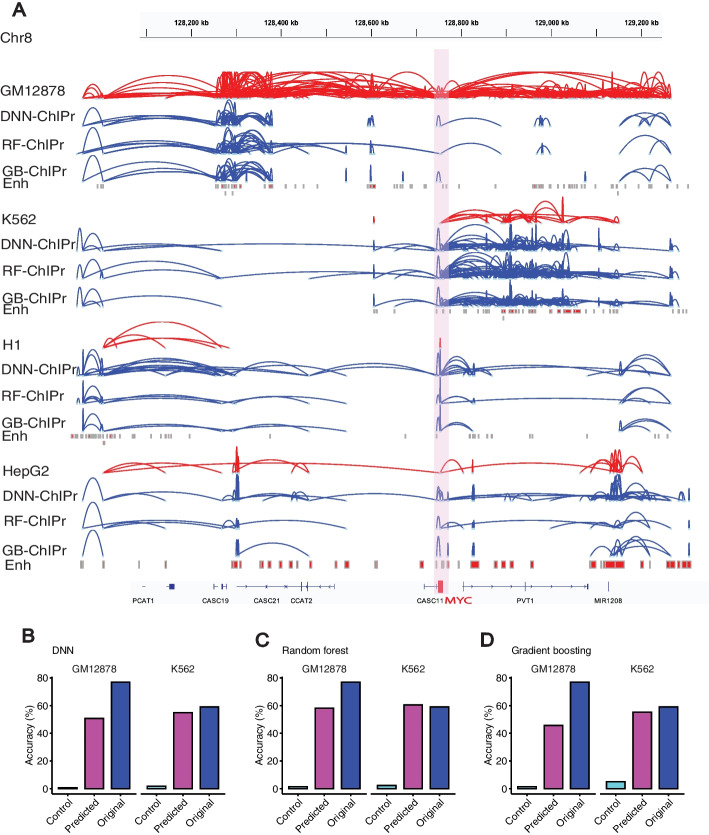
ChIPr captures majority of cell-type-dependent interactions and Hi-C identified loops. **A** Original interactions and predicted ones for the four cell lines GM12878, K562, H1, and HepG2 using the three variants of ChIPr in the region surrounding the *MYC* oncogene. Interactions shown are those having strength ≥ “3”. Red: original loops from RAD21 ChIA-PET data; blue: predicted loops by ChIPr. **B**–**D** Predicted interactions using DNN-ChIPr (**B**), RF-ChIPr (**C**), and GB-ChIPr (**D**) captured the majority of the Hi-C identified loops captured by the original ChIA-PET data from the cell lines GM12878 and K562

Moreover, we also explored loops in the region surrounding the *MYC* oncogene. We found that model predictions could capture the strong interactions between *MYC* promoter and the enhancer elements located in the *PVT1* gene in the four cell lines (Fig. 6A). In addition, the strong set of enhancer-enhancer interactions in the regions of *CASC19* and *CASC21* genes and in the region of *PVT1* gene were also captured by all the three variants of ChIPr in GM12878 and K562 cell lines, respectively (Fig. 6A). We suggest that using all the three ChIPr models is likely to give a more robust view of cohesin-associated chromatin interactions in any region of interest.

### ChIPr captures Hi-C identified cell-type-dependent interactions

In [9], in situ Hi-C was used to investigate the 3D structure of genomes of nine cell types. In addition, HiCCUPS was developed to identify loops in the Hi-C maps. As an independent validation test, we measured the overlap between the strong interactions predicted by ChIPr and the Hi-C identified loops of GM12878 and K562. As a negative control, we also introduced random loops of the same number as the Hi-C identified ones (see Fig. 4A). We found that the predictions of our regression models capture the majority of the loops captured by the original Hi-C data (Fig. 6B–D). We have also found that the Hi-C identified loops captured by the predictions of the three variants of ChIPr are significantly higher than the percentage of randomly introduced loops captured (Additional file 1: Fig. S3A-C). These results suggest that a substantial number of Hi-C loops in these cell types are mediated by cohesin.

### Contributions of input features to the ChIPr predictions

**Fig. 7:**
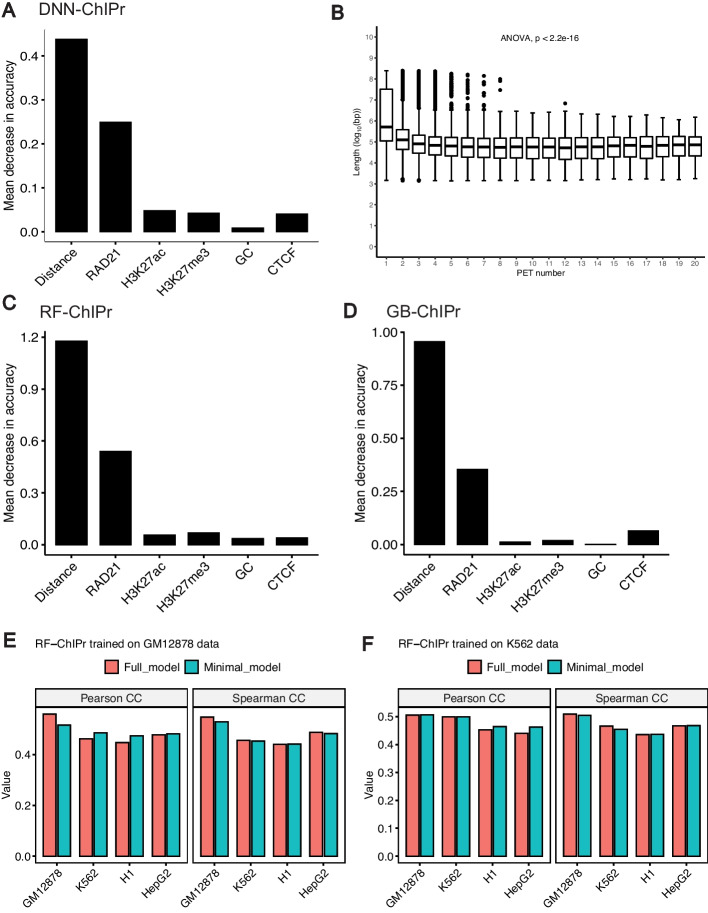
Contributions of input features to ChIPr outputs. **A** The drop in mean absolute error when comparing predicted interactions with the original ones when training DNN-ChIPr while removing one of the input features at each time. **B** The relation between the number of RAD21 interactions with different strengths and the genomic distance between the two interacting peaks. **C**, **D** The importance of the inputs features for RF-ChIPr (**C**) and GB-ChIPr (**D**) using the permutations test. **E**, **F** Comparison between the genome-level performance of RF-ChIPr minimal and full models trained on GM12878 data (**E**) and K562 data (**F**), respectively. The data is split into training data (75%) and test data (25%). In **E**, the performance of GM12878 is measured on the GM12878 test data. Similarly, in **F**, the performance of K562 is also measured on the K562 test data

To measure the importance of each input feature to the prediction accuracy, we trained the DNN-ChIPr model multiple times using the GM12878 data of odd chromosomes, eliminating one of the input features each time. We tested the trained model each time on the data of the even chromosomes and measured the performance according to the mean absolute error value (i.e., the absolute value of the difference between the original value and the predicted one) when compared with the original interactions at the peak-level resolution. Then, we calculated the drop in performance when removing each of the input features (Fig. 7A). We found the largest drop in the performance was due to the removal of the genomic distance input. Hence, we concluded that the genomic distance is the most important of the six input features (this is consistent to the previous ER loop predictor [19]). We also observed an inverse relationship between RAD21 chromatin interaction strength and genomic distance (Fig. 7B). The second most important feature is the interaction mediating protein, RAD21, ChIP-Seq data. Training the model without the H3K27ac, H3K27me3, the GC content of the two interacting peaks, or the CTCF motif orientation flag yielded a very small difference. However, when we removed both H3K27ac and H3K27me3 ChIP-Seq data together, this yielded a slightly bigger drop in performance (Additional file 1: Fig. S4). This shows that, although H3K27ac and H3K27me3 ChIP-Seq signals are anti-correlated, at least one of them should be used in the training of the model. For RF-ChIPr and GB-ChIPr, we used the permutations test (see “Methods” section for more details), and it yielded comparable order of feature importance as for DNN-ChIPr (Fig. 7C, D). These results suggest that training a minimal model with a single experimental data (RAD21 ChIP-Seq data) can produce good-quality prediction results.

### Minimal model with a single experimental data—RAD21 ChiP-Seq

**Fig. 8:**
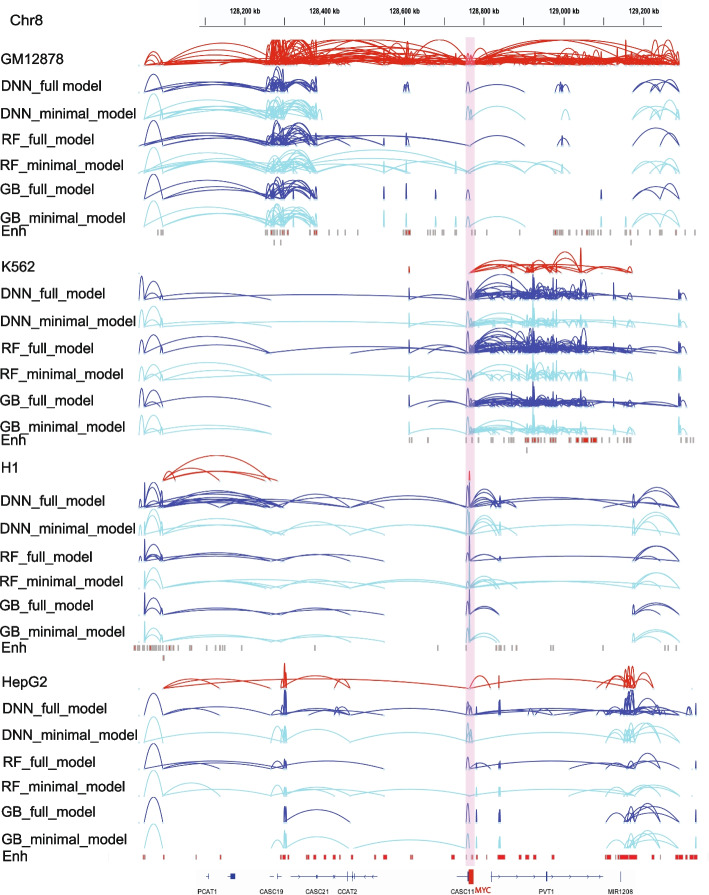
Original interactions and predicted ones for the four cell lines GM12878, K562, H1, and HepG2 using the three variants of ChIPr in the region surrounding the *MYC* oncogene. Interactions shown are those having strength ≥ “3”. Red: original loops from RAD21 ChIA-PET data; blue: predicted loops by ChIPr full model, and cyan: predicted loops by ChIPr minimal model

We tested the utility of training a minimal model using only a single experimental data—RAD21 ChIP-Seq. We trained the three regression models (DNN, Random forest and gradient boosting) with just four input data—RAD21 ChIP-Seq, genomic distance between peaks, GC content and CTCF motif orientation flag. We compared the genome-level performance of the minimal ChIPr model vs. standard six input model (full model). Both models gave comparable results (Fig. 7E, F, Additional file 1: Fig. S5A-D). We also compared the performance of the minimal model with the full model by analyzing the *MYC* locus. Remarkably, both the models performed equally well in predicting the cell-type dependent cohesin-mediated chromatin interactions in the *MYC* locus (Fig. 8).

### The role of CTCF motif, its orientation and CTCF occupancy in cohesin-mediated chromatin interactions

**Fig. 9:**
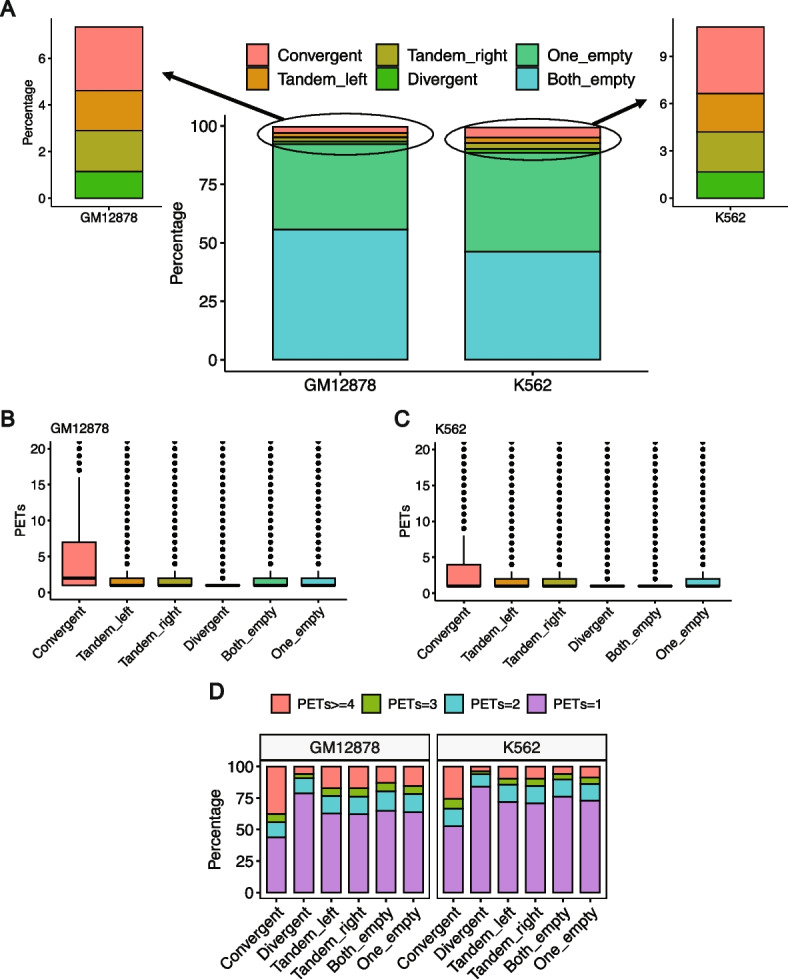
Relationship between RAD21 interactions and CTCF motif orientation. **A** The relationship between the RAD21 interactions prevalence and the presence of CTCF motif in the interacting peaks. **B**, **C** The relationship between the strength of RAD21 interactions and the presence of CTCF motif in the interacting peaks for **B** GM12878 and **C** K562, respectively. **D** The percentage of interactions of different strengths and their relationship with the presence of CTCF motif in the interacting peaks

We analyzed the relationship between the strength and prevalence of the RAD21 interactions with the CTCF motif presence and orientation in the two interacting peak regions in both GM12878 and K562 cell lines. We found that the CTCF motif is found with high confidence in both of the two interacting peaks in ~10% of the RAD21-mediated interactions in the two cell lines (Fig. 9A). In addition, when the CTCF motif is present in both of the two peaks and its orientation is in the convergent manner, the interactions are, on average, stronger than in the other cases, including divergent, tandem left or right, and absence of the motif in one or both peaks (Fig. 9B, C). A big portion of the loops with convergent CTCF motifs (45 and 34% in GM12878 and K562, respectively) exhibit strong interactions (Fig. 9D). However, more than 50% of the interactions are weak (PETs < 3) even with CTCF motif convergent orientation (Fig. 9D). On the other hand, when the CTCF motif orientation is not convergent (divergent, tandem left or right, or the motif does not exist in one or both of the two peaks), we found that more than 70% of the interactions are weak (Fig. 9D). These results show that the convergent CTCF motif orientation is not critical for the strength of the majority of RAD21-mediated interactions, in line with its small contribution to predicting the output of ChIPr (Fig. 7A, C, and D).

**Fig. 10:**
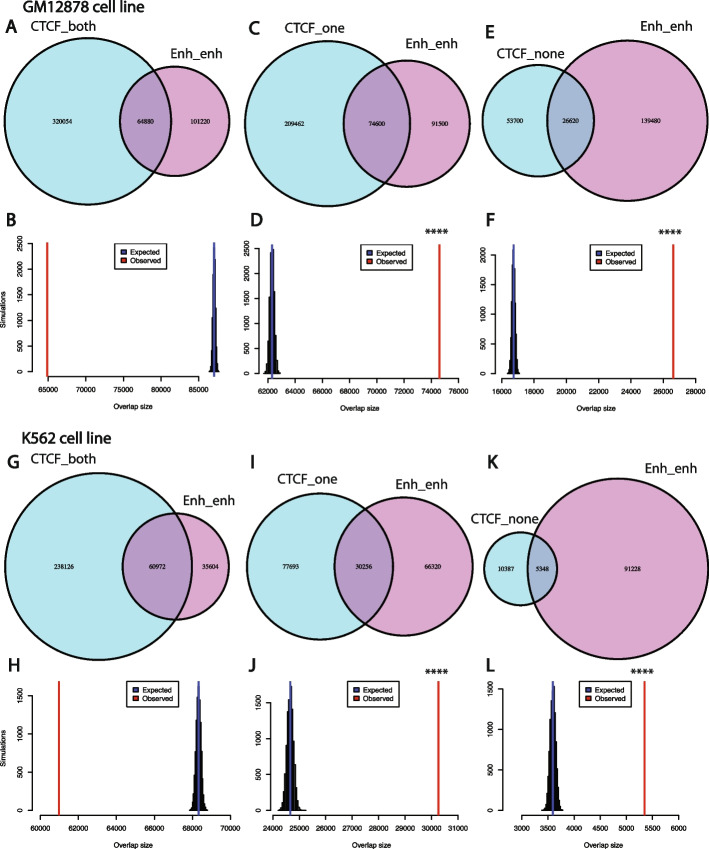
RAD21 interactions without CTCF ChIP-Seq binding in both peaks are significantly enriched with enhancer-enhancer interactions. **A**, **B** Venn diagram showing the intersection between RAD21 interactions with CTCF binding in both peaks with those interactions with enhancer in both peaks for the GM12878 cell line (**A**), and simulations show that the intersection between the two sets of interactions is not significant (**B**). **C**, **D** Venn diagram showing the intersection between RAD21 interactions with CTCF binding in only one peak with those interactions with enhancer in both peaks for the GM12878 cell line (**C**), and simulations show that the intersection between the two sets of interactions is statistically significant (**D**). **E**, **F** Venn diagram showing the intersection between RAD21 interactions with no CTCF binding in both peaks with those interactions with enhancer in both peaks for the GM12878 cell line (**E**), and simulations show that the intersection between the two sets of interactions is statistically significant (**F**). **G**–**L** Same as **A**–**F** for the K562 cell line. ****: *p*-value < 0.0001, empirical test

**Fig. 11:**
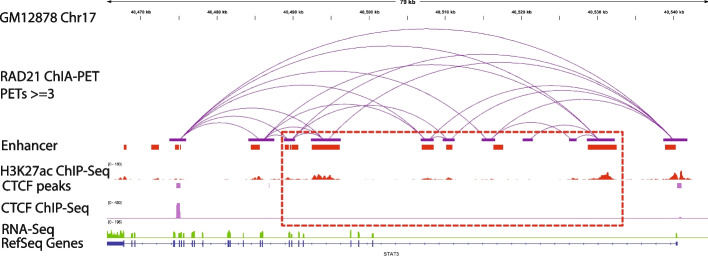
Example of RAD21 interactions without CTCF in the two anchor peaks and are found to be enhancer-enhancer interactions in the GM12878 cell line (middle track in purple)

In addition, we analyzed the relationship between RAD21 interactions and CTCF ChIP-seq peaks. This analysis showed that ~50–73% of the RAD21 interactions were enriched with CTCF binding (CTCF ChIP-Seq peaks) in the two anchor peak regions of the interaction. However, less than 15% of the interactions had no CTCF ChIP-seq binding in both of the two peaks. RAD21 interactions that either lack CTCF binding in both the anchors or one of the two anchors were significantly enriched for enhancer-enhancer interactions (see “Methods”, Additional file 1: Figs. S6-S8; Fig. 10). For example, in GM12878 cells, the *STAT3* locus harbors multiple cohesin-mediated chromatin interactions forming an insulated neighborhood (Fig. 11). The outer RAD21 interactions in the locus are anchored by CTCF, whereas the inner RAD21 interactions are not anchored by CTCF. These inner RAD21 interactions represent enhancer-enhancer interactions. We also extended the analysis to three additional cell lines whose RAD21 ChIA-PET data and CTCF ChIP-Seq data are both available (H9, MCF7, and LNCaP). We found that RAD21 interactions without CTCF binding in both peaks in these three cell lines are also significantly enriched with enhancer-enhancer interactions (Additional file 1: Figs. S9-S12). Taken together, these results suggest that CTCF motif presence is not a common feature of all cohesin-mediated chromatin interactions. However, CTCF occupancy is a common—but not a universal feature—of cohesin-mediated chromatin interactions. There can be multiple explanations for the discrepancy between the CTCF motif and CTCF occupancy in cohesin-mediated chromatin interactions. There could be weak or variant CTCF binding sites below our motif detection level. Indeed when we performed motif enrichment analysis for the peaks where CTCF binds without the presence of the CTCF motif in the GM12878 cell line using HOMER [34], we found that, in these locations, other variants of the CTCF motifs with several alignment mismatches are significantly enriched (Additional file 1: Fig. S13). In addition, it has been shown that, in general, transcription factor binding may occur in the absence of any discernible motif instance, or it may occur at “hotspots” where several factors are found together [35].

Moreover, we have analyzed the interaction strength of RAD21 interactions with CTCF ChIP-Seq binding in both peaks and other RAD21 interactions (with CTCF binding in one peak or without CTCF binding in both peaks). We have found that interactions with CTCF binding in both peaks are generally significantly stronger than the other two classes (Additional file 1: Fig. S14). This is consistent with the important role of these interactions in forming domain boundaries and insulated neighborhoods [36].

### ChIPr outperforms other 3D chromatin interaction prediction methods

**Fig. 12:**
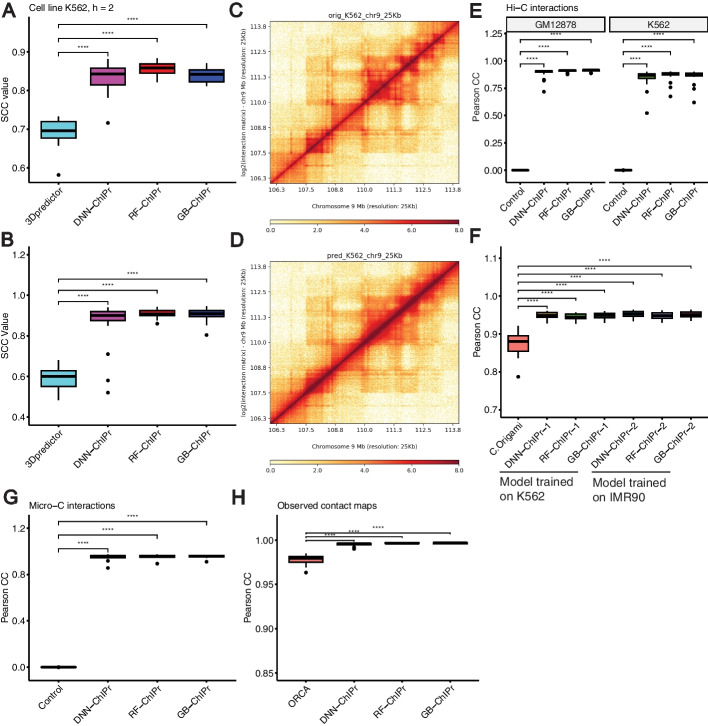
ChIPr outperforms other 3D chromatin prediction methods. **A** The RAD21 25 kbp contact maps predicted by the three variants of ChIPr yield significantly higher SCC values when compared with the original contact maps than those yielded by 3Dpredictor contact maps. **B** The 25 kbp Hi-C contact maps predicted by the three variants of ChIPr yield significantly higher SCC values when compared with the original contact maps than those yielded by 3Dpredictor contact maps. **C**, **D** The original contact map of an 8 Mbp region in Chr9 (**C**) looks visually similar to its corresponding GB-ChIPr (**D**) prediction. **E** The predictions of the three variants of ChIPr, using the models trained on the IMR90 cell line data, correlate significantly better than control interactions when compared with the original Hi-C interactions for the two cell lines GM12878 and K562. **F** The predictions of the three variants of ChIPr, using the models trained on K562 and IMR90 cell line data, yield significantly higher Pearson correlation coefficient values than those obtained by C.Origami when comparing the 8 kbp resolution contact maps with the corresponding original ones for 23 randomly selected 2Mbp regions. **G** The predictions of the three variants of ChIPr, using the models trained on the H1 cell line Micro-C data (Chr1 only), correlate significantly better than control interactions when compared with the original Micro-C interactions for the remaining 22 chromosomes. **H** The three variants of ChIPr yield significantly higher Pearson correlation coefficient values when comparing the 5 kbp resolution observed contact maps with the original contact maps than those yielded by Orca 4 kbp contact maps for 16 selected regions in Chrs 8, 9, and 10. ****: *p*-value < 0.0001, Wilcoxon rank sum test. For SCC calculations in **A**, **B**, we set the smoothing window half-size *h* to “2” and the maximum genomic distance to include in calculations to 1.5 Mbp as used in 3Dpredictor paper. For **C**, **D**, visualization of contact maps is done using HiCPlotter [37]

Since there are no other tools for predicting cohesin-mediated interactions’ strength, we compared ChIPr to another tool originally used to predict Hi-C interactions. 3Dpredictor can predict Hi-C contact maps at relatively high resolutions like 5 and 25 kbp bins using genomic distance, CTCF binding information, and RNA-seq data as inputs [22]. To use 3Dpredictor to predict cohesin-mediated interactions, we constructed 25-kbp contact maps of the K562 cell line using its original RAD21 ChIA-PET interactions. We used the contact maps of odd chromosomes to train 3Dpredictor and predicted the contact maps of even chromosomes. We also trained the three variants of ChIPr using the K562 cell line odd chromosomes and predicted the interactions of even chromosomes at the peak-level resolution using ChIPr. To compare the outputs of both methods, we constructed the contact maps of even chromosomes using the predictions of ChIPr at 25 kbp resolution. The three variants of ChIPr significantly outperformed 3Dpredictor according to stratum-adjusted correlation coefficient (SCC) (Fig. 12A).

Also, we trained ChIPr with K562 Hi-C data (Chr1) at 5 kbp resolution. We predicted the Hi-C interactions of the other 22 chromosomes at 5 kbp resolution, and we generated 25 kbp resolution contact maps with these predictions. We also trained 3Dpredictor with K562 Hi-C data of Chr1 at 25 kbp resolution and predicted the contact maps of the other 22 chromosomes. We chose the K562 cell line because its training data for 3Dpredictor is available on the GitHub page of the method (https://github.com/labdevgen/3Dpredictor/tree/master). We compared the contact maps generated by both ChIPr and 3Dpredictor to the original contact maps of the K562 cell line. We found that the three variants of ChIPr significantly outperformed 3Dpredictor according to SCC (Fig. 12B). We also found the predicted Hi-C maps using ChIPr are visually similar to the original Hi-C maps (Fig. 12C, D). This further indicates the outperformance of ChIPr over 3Dpredictor and shows the validity of ChIPr to predict Hi-C data in addition to cohesin-mediated interactions’ strengths.

In addition, several methods were developed recently to predict Hi-C-like contact maps at high resolution for sub-megabase genomic distances like Orca [26] and C.Origami [25]. While Orca predicts chromatin interactions based on genomic distance and sequence only, C.Origami also uses two cell-type-specific features: CTCF ChIP-Seq data and ATAC-Seq data. To compare ChIPr with C.Origami and Orca, we used the data they used to measure their performances. More specifically, we trained the three variants of ChIPr using IMR90 cell line Hi-C data using Chr1 only for training. We used the trained models to predict Hi-C interactions of the two cell lines, K562 and GM12878. We found that the predictions correlate with the original interactions of the two cell lines significantly better than control interactions (see “Methods”) (Fig. 12E). Then, for randomly selected 2-Mb regions (see Additional file 2: Table S1), we predicted 8 kbp resolution contact maps for the GM12878 cell line using models trained on K562 and IMR90 cell lines data, respectively. We also used C.Origami to predict the GM12878 contact maps of the same regions using a model trained on IMR90 cell line data. For comparison, we calculated the logged Hi-C intensity with iterative correction and eigenvector decomposition (ICE): (log(ICE normalized counts + 1)) for the contact maps predicted using ChIPr and the original Hi-C contact maps for the selected regions as suggested by C.Origami [25]. We found that the predictions of the three variants of ChIPr using the two models trained on K562 and IMR90 cell lines data, respectively, correlate significantly higher with the original contact maps than the predictions of C.Origami (Fig. 12F). These results show that ChIPr can be used to predict Hi-C data efficiently and can outperform C.Origami.

In addition, we compared ChIPr with Orca using the data they used to measure their performance. We trained ChIPr on the Micro-C data of the H1 cell line [38]. We used only Chr1 for training, and we tested the performance of ChIPr in predicting the other 22 chromosomes of the same cell line. We found that the predictions of the three variants of ChIPr correlate significantly better with the original interactions than control interactions (see “Methods”) (Fig. 12G). We also constructed 1 Mb contact maps at 5 kbp resolution for several regions in Chrs 8, 9, and 10 (the chromosomes used for validation and testing of the Orca-trained model). We predicted the contact maps of the same regions (see Additional file 2: Table S2) using Orca (https://orca.zhoulab.io/) at 4 kbp resolution. For comparison, we performed the same pre-processing step suggested by Orca on ChIPr predictions and the corresponding original contact maps, iterative correction matrix balancing algorithm and adaptive coarse graining [26]. Although the performance of both methods was very high, probably because we are testing using hold-out chromosomes of the same cell line used for training, we found that Pearson correlation values of the original maps with ChIPr predictions are significantly higher than those with Orca predictions (Fig. 12H). All these results show the outperformance of ChIPr over other 3D chromatin prediction methods. These results also indicate that ChIPr can efficiently predict high-resolution Micro-C contact maps (5 kbp resolution).

## Discussion

In this study, we present ChIPr, three regression models based on DNN, random forest, and gradient boosting, respectively, and predict the strength of RAD21-mediated chromatin interactions at the peak-level resolution. ChIPr uses a few input ChIP-Seq samples and other easily obtainable public data for training, testing, and prediction. We have shown that the most important feature for predicting a functional cohesin loop is the genomic distance (loop length), in line with previous report for predicting ER loops [19]. The second most important feature was the ChIP-Seq data for the interaction mediating protein (which was RAD21 in all our analyses), consistent with the expected detection of ChIP-Seq peaks of the mediating protein at the interacting loci regions [39]. However, we found much less importance for the two histone mark profiles, H3K27ac and H3K27me3. This may be due to the fact that these two histone marks are anti-correlated. Thus, the presence of only one of them is enough to get high prediction accuracy. When both of them were removed, we noticed a slightly bigger drop in the prediction accuracy in some cases. However, in general, the results were still very comparable. We also noticed a very small contribution from the GC content information of the two interacting peaks and the CTCF motif convergence flag. A detailed analysis of CTCF motif presence and orientation with the RAD21 interactions prevalence and strength indicated that CTCF motif presence is not necessary for RAD21 interactions’ prevalence. However, its presence and convergent orientation are associated in ~30–40% of the cases with strong RAD21 interactions. We have also observed the occupancy of CTCF in both of the two peaks in most of the RAD21 interactions. In the absence of CTCF binding, we found that RAD21 loops are significantly enriched with enhancer-enhancer interactions.

We have shown that the RAD21-mediated DNA loop prediction outputs of ChIPr correlate well with the original RAD21 ChIA-PET data at the peak-level resolution. They also correlate well at the resolution of bin sizes 25 and 5 kbp, which suggests that we can reliably use ChIPr predictions to detect TAD boundaries. We have also demonstrated that ChIPr could capture most of the ChIA-PET- and Hi-C-identified cell-type-dependent loops as strong interactions. In addition, we have shown that ChIPr outperforms other 3D chromatin interaction prediction methods and that its utility can be extended to predict high-resolution Hi-C and Micro-C contact maps. Altogether, we have shown multiple lines of evidence that ChIPr could reliably reproduce much of the ChIA-PET information using a minimal number of easily obtainable features. These studies outline the general features of genome folding and open new avenues to analyze the spatial genome organization in specimens with limited cell numbers.

Finally, the main purpose of ChIPr is to estimate cohesin-mediated interaction strength between anchor peaks when interaction data, such as ChIA-PET data, is unavailable. We recommend that the three models of ChIPr be used side by side to confirm whether the interaction strength prediction is strong or weak. If all three regression methods of ChIPr predict strong interaction (interaction strength ≥ 3) between two anchor peaks, it is likely that the interaction between these two peaks is truly strong. Similarly, if the three of them predict weak interactions (interaction strength < 3), it is likely that the interaction is truly weak.

## Conclusions

In this study, we present ChIPr, a suite of regression models to predict the strength of cohesin-mediated interactions. We have shown that ChIPr can accurately predict the interactions’ strength at peak resolution and bin sizes of 5 and 25 kbp. We have also demonstrated that ChIPr can predict most of the cell-type-specific loops identified by ChIA-PET or Hi-C as strong interactions. In addition, we report a class of cohesin-mediated interactions that lack CTCF binding and are significantly enriched for enhancer-enhancer interactions. Moreover, we have shown that ChIPr can predict Hi-C and Micro-C interactions and outperform other chromatin prediction methods.

## Methods

### Neural networks, random forest, and gradient boosting

A neural network (NN) [40], in its simplest form, is a system that consists of an input layer, one or more hidden layers, and an output layer. Each layer is connected to the following layer through edges, and each edge has a certain weight. The neural network model is trained by providing many input/output examples. The model learns by gradually changing the edges’ weights between layers until the NN model’s output is very close to the desired output given in the training examples.

Both random forest [41] and gradient boosting [42] are decision tree-based regression models. In random forest, the training data is divided into *N* subsamples with repetition. For each subsample of training data, a decision tree is built to learn the relation between inputs and outputs in this subsample. Thus, we will have an ensemble of *N* decision trees, and the final decision is obtained by majority voting.

On the other hand, in gradient boosting, the training starts with a random guess for the output. Then, the error, which is the difference between the output of the model and the actual desired output, is calculated. We then keep growing decision trees to the model to minimize the calculated error.

### Structure of ChIPr

ChIPr is composed of three variants of regression models based on DNN, random forest, and gradient boosting, respectively. ChIPr uses six input features of the two interacting peaks to predict the RAD21-mediated interactions’ strengths. The first input feature is the linear genomic distance between the centres of the two peaks in kilobases. We have chosen the genomic distance because it is known to be a good predictor of the interaction strength, and it is usually inversely proportional with it according to both Hi-C and fluorescence in situ hybridization (FISH) experiments [3, 43]. The second input feature is the RAD21 ChIP-Seq data at the two interacting peaks. It is expected that two peaks will be detected by the RAD21 ChIP-Seq data at the two interacting loci [39]. In addition, we use the ChIP-Seq data at the two peaks for two canonical histone modification marks, H3K27ac and H3K27me3, which should correlate with active and inactive chromatin states, respectively [9].

Moreover, it is known that the human genome is organized into long (> 300 kbp), relatively homogeneous regions called isochores, which differ in their GC content [44]. It has also been reported that 66% of the genes are present in the GC-rich and GC-richest isochores [44], suggesting a relation between gene distribution and the GC level. Accordingly, we sought that there may be a relation between chromatin activity (which leads to strong interactions) and GC content as well. Thus, we used the GC content of the two peaks as the following two input features to our regression model. Besides, it was reported in [9] that for the Hi-C-identified loops whose corresponding anchor loci contain the CTCF motif, most of the motif pairs are convergent. Thus, we added an input that denotes the convergence of the CTCF motif orientation in the two peaks. This input is “1” if the CTCF motif orientation is convergent. If the CTCF motif orientation in the two peaks is divergent, tandem left or right, or if the motif is absent in one or both peaks, the CTCF motif orientation input will be “0”.

### Hyper-parameter selection for DNN-ChIPr

To decide the architecture of DNN-ChIPr, we used grid search to determine the best number of layers, number of neurons in each layer, dropout rate, batch size, and activation function for the output layer. We have fixed another set of hyper-parameters that are commonly used. For instance, we fixed the activation function for the hidden layers to be “relu” [45]. We have also used the “Adam” optimizer [46] with a small learning rate of 10^−5^. We selected this small learning rate, although it will require a relatively longer training time to ensure the stability of the training process. In addition, we used a large number of epochs (750), with early stopping if no improvement in performance (using the validation mean squared error metric) is observed for 50 epochs. The performance of each model was measured according to the mean squared error loss on the validation data. We found several models gave very comparable values of validation mean squared error (Additional file 3: Table S3). We chose our final model to have three hidden layers; each has 128 neurons, with “relu” activation function for the output node (to ensure that the output is always bigger than zero) and values of 0.2 and 32 for the dropout rate and the batch size, respectively (Additional file 3: Table S3).

### Preparation of the training data

The ChIA-PET data of the four cell lines GM12878, K562, H1, and HepG2 were downloaded from the ENCODE project [12]. The data was processed using the ChIA-PET2 pipeline [47] to get the inter- and intra-chromosomal interactions files. We focused on the intra-chromosomal interactions, and for each interaction, we got the coordinates of the two anchor peaks and the interaction strength. We calculated the input features of anchor peaks of each interaction which comprise alongside the interaction strength a training example to ChIPr. We divide the interactions of the cell line used in training into 75% (training set) and 25% (testing set) and calculate the model’s performance on the testing set. In addition, we test the model on the data of the other three cell lines. For instance, if we train the model using GM12878 data, we test the model on the 25% portion of the GM12878 data (testing set) and the data of the three cell lines K562, H1, and HepG2.

### ChIP-Seq data normalization

We used the RAD21, H3K27ac, and H3K27me3 ChIP-Seq data of our four investigated cell lines (GM12878, K562, H1, and HepG2). We calculated the read count for each of the two anchor peaks of each loop. To account for the sample’s sequencing depth and the peaks’ sizes, we normalized the ChIP-Seq data using the reads per kilobase per million (RPKM) normalization method, described in the following few lines. We first get the “per million scaling factor” by dividing the total number of reads in the chromosome by 1,000,000. Then, we divide the read count in each peak by the “per million scaling factor”, a step that accounts for the effect of sequencing depth. After that, we divide by the peak length in kilobases, a step that accounts for the peak length.

### Obtaining the GC content and CTCF motif orientation within peaks

To include sequence information into our model, we calculated the GC content for each peak, defined as the percentage of cytosine (C) and guanine (G) bases in that peak. We calculated it using the bedtools nuc function [48].

Also, we used GimmeMotifs [49] and CTCF Bioconductor package [50] to detect the presence of the CTCF motif in each peak and its orientation. GimmeMotifs reports only one motif occurrence per peak (the highest scoring motif). For the CTCF motif orientation input, we set it to “1” if the CTCF motif orientation in the two peaks is convergent and “0” otherwise.

### Constructing contact maps from peak-level interactions

ChIPr predicts interaction strength between peaks. The peak length is in the range of 2 kbp. It may be slightly smaller or larger than that. From interactions between peaks, we can build bin-based contact maps. We do that by summing all interactions whose anchor peaks lie within the same bin. For instance, to build a 25-kbp-bin contact map, we have a square matrix where each entry represents the interaction strength between two 25-kbp bins. To get the value of interaction strength between a pair of bins, we sum all the interactions whose anchor peaks fall within these two bins.

### Testing the model performance at the peak-level resolution

For each interaction in the test set, we have the coordinates of the two anchor peaks of the interaction. We calculate the input features required by ChIPr for the two peaks, and then we use ChIPr to get the predicted output for that particular interaction. We also generate a control interaction strength for the same interaction (a random value drawn from a negative binomial distribution having the same mean and variance as the original interactions in the original ChIA-PET file). Thus, for all interactions in the test set, we have the predicted outputs and the control values. We measure the correlation between them and the original values from the original ChIA-PET file to evaluate the performance of the model at the peak-level resolution.

### Permutation test for determining feature importance

To calculate the permutation importance of a certain feature, a baseline metric (for instance, mean absolute error) is evaluated on the test data (for instance, the data of even chromosomes if the training was done using the data of odd chromosomes). Then, for the feature column that is required to measure its importance, this column is permuted, and the metric is evaluated again. The permutation importance of that feature is defined as the difference between the baseline metric and the metric obtained after the permutation of the feature column [41].

### Analysis of CTCF occupancy in cohesin-mediated interactions

We get the CTCF ChIP-Seq peak files of each cell line, and use bedtools intersect function to determine if CTCF peaks overlap with none, one, or both of the two anchor peaks of the RAD21 interactions.

### Empirical test to calculate the overlap significance (*p*-value)

To calculate the *p*-value of the intersection between enhancer-enhancer interactions and different classes of interactions (interactions with CTCF in both peaks, in one peak only, or in none of the two peaks), we used an empirical test as described here. First, we get the actual “observed” value of the intersection between the two sets of interactions. Then, we calculate the “expected” value of the intersection as follows. We initially set a counter to zero and perform the following simulation 10,000 times. From the whole list of RAD21 interactions, we sample a random set of interactions with the same number as the number of enhancer-enhancer interactions. We calculate the intersection between the randomly sampled list with the class of interactions of interest (e.g., interactions with CTCF in both peaks). If the intersection value is bigger than the “observed” value calculated above, we increment the counter by “1”. After the 10,000 simulations, the empirical *p*-value will be = counter/10,000. And the “expected” value of overlap is the average of the calculated intersection in the 10,000 simulations.

### Generating control interactions for Hi-C and Micro-C data

To test the performance of predicting Hi-C and Micro-C data, we generated control interactions for comparison purposes. To generate the control interactions, we shuffled the input features of the chromosome of interest and used these shuffled data as inputs to the ChIPr-trained models. The outcome of this step, predicted Hi-C and Micro-C interactions for shuffled inputs, was compared to the original outputs with the same original order to obtain the Pearson correlation value of the control interactions. This ensures that the control interactions are produced using input features with the same properties, especially the same genomic distance distribution, as those used to obtain ChIPr predictions.

### Implementation details

The DNN model (DNN-ChIPr) was implemented using Keras, the Python deep learning API (https://keras.io/). For the random forest (RF-ChIPr) and gradient boosting (GB-ChIPr) models, we used sklearn RandomForestRegressor and GradientBoostingRegressor with the default parameters, respectively [51].

### Supplementary Information

**Additional file 1: Fig. S1.** Predicted interactions correlate well with the original ones at the peak-level resolution. (A) Predicted interactions using the three variants of ChIPr for the cell lines K562, H1, and HepG2 correlate significantly better than the random interactions with the original ChIA-PET interactions of these three cell lines according to Spearman correlation coefficient. The predictions in (A) are obtained using models trained on the GM12878 cell line data. (B) Predicted interactions using the three variants of ChIPr for the cell lines GM12878, H1, and HepG2 correlate significantly better than the random interactions with the original ChIA-PET interactions of these three cell lines according to Spearman correlation coefficient. The predictions in (B) are obtained using the models trained on the K562 cell line data. ****: *p*-value < 0.0001, Wilcoxon rank sum test. **Fig. S2.** Predicted interactions correlate well with the original ones at the 5 Kbp bin resolution. (A and B) Comparison between the correlation coefficient values between the original interactions and the predicted ones using the three variants of ChIPr vs. those between the original and randomly generated ones for the four cell lines GM12878, K562, H1, and HepG2. The correlation coefficients were calculated using stratum adjusted correlation coefficients (A) and Pearson correlation coefficients (B), respectively. Predictions for cell lines GM12878, H1, and HepG2 were calculated using the models trained on K562 data. Predictions for the cell line K562 were calculated using the models trained on GM12878 data. **Fig. S3.** ChIPr predictions (DNN-ChIPr (A), RF-ChIPr (B), and GB-ChIPr (C)) capture Hi-C identified loops at significantly higher percentage than control loops. ****: *p*-value < 0.0001, Wilcoxon rank sum test. **Fig. S4.** The drop in mean absolute error when comparing predicted interactions with the original ones when training DNN-ChIPr while removing one of the input features at each time. The plot shows that removing H3K27ac and H3K27me3 together causes a relatively bigger drop in performance than removing each of them alone. **Fig. S5.** Comparison between the genome-level performance of minimal and full models of DNN-ChIPr (A and B) and GB-ChIPr (C and D). The models in (A and C) were trained on the data of GM12878 cell line. The models in (B and D) were trained on the data of K562 cell line. The data is split into training data (75%) and test data (25%). In (A and C), the performance of GM12878 is measured on the GM12878 test data. Similarly, in (B and D), the performance of K562 is also measured on the K562 test data. **Fig. S6.** The majority of RAD21 interactions have CTCF ChIP-seq binding in both of the two anchor peaks of the interactions in the four cell lines GM12878, K562, H1, and HepG2. The portion of interactions that misses CTCF ChIP-seq binding in the two anchor peaks is mostly enriched with enhancer-enhancer interactions. **Fig. S7.** RAD21 interactions without CTCF ChIP-seq binding in both peaks are significantly enriched with enhancer-enhancer interactions. (A and B) Venn diagram showing the intersection between RAD21 interactions with CTCF binding in both peaks with those interactions with enhancer in both peaks for the H1 cell line (A), and simulations show that the intersection between the two sets of interactions is not significant (B). (C and D) Venn diagram showing the intersection between RAD21 interactions with CTCF binding in only one peak with those interactions with enhancer in both peaks for the H1 cell line (C), and simulations show that the intersection between the two sets of interactions is statistically significant (D). (E and F) Venn diagram showing the intersection between RAD21 interactions with no CTCF binding in both peaks with those interactions with enhancer in both peaks for the H1 cell line (E), and simulations show that the intersection between the two sets of interactions is statistically significant (F). ****: *p*-value < 0.0001, empirical test. **Fig. S8.** RAD21 interactions without CTCF ChIP-seq binding in both peaks are significantly enriched with enhancer-enhancer interactions. (A and B) Venn diagram showing the intersection between RAD21 interactions with CTCF binding in both peaks with those interactions with enhancer in both peaks for the HepG2 cell line (A), and simulations show that the intersection between the two sets of interactions is not significant (B). (C and D) Venn diagram showing the intersection between RAD21 interactions with CTCF binding in only one peak with those interactions with enhancer in both peaks for the HepG2 cell line (C), and simulations show that the intersection between the two sets of interactions is statistically significant (D). (E and F) Venn diagram showing the intersection between RAD21 interactions with no CTCF binding in both peaks with those interactions with enhancer in both peaks for the HepG2 cell line (E), and simulations show that the intersection between the two sets of interactions is statistically significant (F). ****: *p*-value < 0.0001, empirical test. **Fig. S9.** The majority of RAD21 interactions have CTCF ChIP-seq binding in both of the two anchor peaks of the interactions in the three cell lines H9, MCF7, and LNCaP. The portion of interactions that misses CTCF ChIP-seq binding in the two anchor peaks is mostly enriched with enhancer-enhancer interactions. **Fig. S10.** RAD21 interactions without CTCF ChIP-seq binding in both peaks are significantly enriched with enhancer-enhancer interactions. (A and B) Venn diagram showing the intersection between RAD21 interactions with CTCF binding in both peaks with those interactions with enhancer in both peaks for the H9 cell line (A), and simulations show that the intersection between the two sets of interactions is not significant (B). (C and D) Venn diagram showing the intersection between RAD21 interactions with CTCF binding in only one peak with those interactions with enhancer in both peaks for the H9 cell line (C), and simulations show that the intersection between the two sets of interactions is statistically significant (D). (E and F) Venn diagram showing the intersection between RAD21 interactions with no CTCF binding in both peaks with those interactions with enhancer in both peaks for the H9 cell line (E), and simulations show that the intersection between the two sets of interactions is statistically significant (F). ****: *p*-value < 0.0001, empirical test. **Fig. S11.** RAD21 interactions without CTCF ChIP-seq binding in both peaks are significantly enriched with enhancer-enhancer interactions. (A and B) Venn diagram showing the intersection between RAD21 interactions with CTCF binding in both peaks with those interactions with enhancer in both peaks for the MCF7 cell line (A), and simulations show that the intersection between the two sets of interactions is not significant (B). (C and D) Venn diagram showing the intersection between RAD21 interactions with CTCF binding in only one peak with those interactions with enhancer in both peaks for the MCF7 cell line (C), and simulations show that the intersection between the two sets of interactions is statistically significant (D). (E and F) Venn diagram showing the intersection between RAD21 interactions with no CTCF binding in both peaks with those interactions with enhancer in both peaks for the MCF7 cell line (E), and simulations show that the intersection between the two sets of interactions is statistically significant (F). ****: *p*-value < 0.0001, empirical test. **Fig. S12.** RAD21 interactions without CTCF ChIP-seq binding in both peaks are significantly enriched with enhancer-enhancer interactions. (A and B) Venn diagram showing the intersection between RAD21 interactions with CTCF binding in both peaks with those interactions with enhancer in both peaks for the LNCaP cell line (A), and simulations show that the intersection between the two sets of interactions is not significant (B). (C and D) Venn diagram showing the intersection between RAD21 interactions with CTCF binding in only one peak with those interactions with enhancer in both peaks for the LNCaP cell line (C), and simulations show that the intersection between the two sets of interactions is statistically significant (D). (E and F) Venn diagram showing the intersection between RAD21 interactions with no CTCF binding in both peaks with those interactions with enhancer in both peaks for the LNCaP cell line (E), and simulations show that the intersection between the two sets of interactions is statistically significant (F). ****: *p*-value < 0.0001, empirical test. **Fig. S13.** HOMER results for the top enriched motif (BORIS) and its top four best matches with known motifs in the locations of CTCF binding for the GM12878 cell line. **Fig. S14.** RAD21 interactions with CTCF ChIP-Seq binding in both peaks are significantly stronger than those with CTCF binding in one peak only or in none of the two peaks.

**Additional file 2: Table S1 and Table S2.** They contain the regions tested in comparison with other chromatin interaction prediction methods.

**Additional file 3: Table S3.** It contains the results obtained for the grid search for the DNN hyperparameter selection.

**Additional file 4: Table S4.** It contains a summary of the datasets used and their accession numbers.

**Additional file 5.** Review history.

## Data Availability

Datasets used in this study are all publicly available. The RAD21 ChIA-PET data for the four cell lines GM12878, K562, H1, and HepG2 can be downloaded from the ENCODE portal [12]. The RAD21 ChIP-Seq data for the four cell lines can be downloaded from NCBI GEO: GSM935332 (GM12878 cell line), GSM935319 (K562 cell line), GSM935379 (H1 cell line), and GSM935647 (HepG2 cell line) [29]. The H3K27ac ChIP-Seq data for the four cell lines can be downloaded from NCBI GEO: GSM733771 (GM12878 cell line), GSM733656 (K562 cell line), GSM733718 (H1 cell line), and GSM733743 (HepG2 cell line) [29]. The H3K27me3 ChIP-Seq data for the four cell lines can be downloaded from NCBI GEO: GSM733758 (GM12878 cell line), GSM733658 (K562 cell line), GSM733748 (H1 cell line), and GSM733754 (HepG2 cell line). The CTCF ChIP-Seq peaks can be downloaded from NCBI GEO: GSM935611 (GM12878 cell line), GSM935407 (K562 cell line), GSM733672 (H1 cell line), GSM733645 (HepG2 cell line), GSE105500 (LNCaP cell line), GSE175278 (H9 cell line), and GSM1006878 (MCF7 cell line). The Hi-C data of the three cell lines GM12878, K562, and IMR90 can be downloaded from GSE63525 [9]. The Micro-C data of the H1 cell line can be downloaded from the 4DN data portal with the accession number 4DNFI9GMP2J8 [38]. Enhancer lists for the four cell lines can be downloaded from EnhancerAtlas 2.0 [52] (http://www.enhanceratlas.org/indexv2.php). A summary of the data used can also be found in Additional file 4: Table S4. Source code of ChIPr with a detailed Readme file is freely available under the GNU General Public License Version 2 (GPLv2) at https://git.biohpc.swmed.edu/s206442/chipr [53]. It has also been deposited at Zenodo with DOI 10.5281/zenodo.10364968 [54].
