# Supplementary material for: ChIPr: accurate prediction of cohesin-mediated 3D genome organization from 2D chromatin features
Source: Genome Biol. 2024 Jan 12;25:15. doi: 10.1186/s13059-023-03158-7 (PMC10785520; doi:10.1186/s13059-023-03158-7)
Supplement: Supplementary file 1 — Additional file 1: Fig. S1. Predicted interactions correlate well with the original ones at the peak-level resolution. (A) Predicted interactions using the three variants of ChIPr for the cell lines K562, H1, and HepG2 correlate significantly better than the random interactions with the original ChIA-PET interactions of these three cell lines according to Spearman correlation coefficient. The predictions in (A) are obtained using models trained on the GM12878 cell line data. (B) Predicted interactions using the three variants of ChIPr for the cell lines GM12878, H1, and HepG2 correlate significantly better than the random interactions with the original ChIA-PET interactions of these three cell lines according to Spearman correlation coefficient. The predictions in (B) are obtained using the models trained on the K562 cell line data. ****: p-value < 0.0001, Wilcoxon rank sum test. Fig. S2. Predicted interactions correlate well with the original ones at the 5 Kbp bin resolution. (A and B) Comparison between the correlation coefficient values between the original interactions and the predicted ones using the three variants of ChIPr vs. those between the original and randomly generated ones for the four cell lines GM12878, K562, H1, and HepG2. The correlation coefficients were calculated using stratum adjusted correlation coefficients (A) and Pearson correlation coefficients (B), respectively. Predictions for cell lines GM12878, H1, and HepG2 were calculated using the models trained on K562 data. Predictions for the cell line K562 were calculated using the models trained on GM12878 data. Fig. S3. ChIPr predictions (DNN-ChIPr (A), RF-ChIPr (B), and GB-ChIPr (C)) capture Hi-C identified loops at significantly higher percentage than control loops. ****: p-value < 0.0001, Wilcoxon rank sum test. Fig. S4. The drop in mean absolute error when comparing predicted interactions with the original ones when training DNN-ChIPr while removing one of the input features at each time. [file 13059_2023_3158_MOESM1_ESM.pdf]

Fig. S1

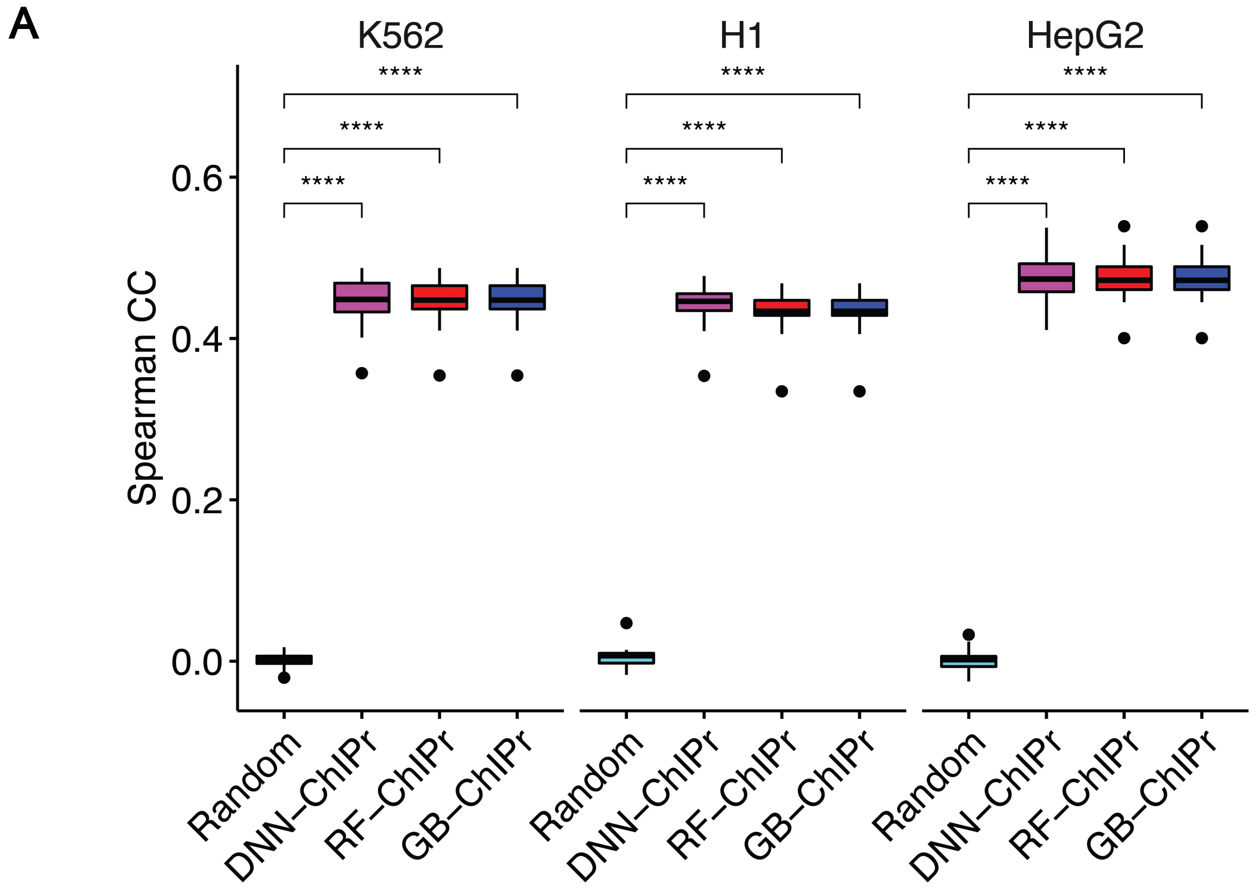
A
K562
H1
HepG2
****
****
****
****
****
****
****
****
****
Spearman CC
0.6
0.4
0.2
0.0
Random
DNN–ChIPr
RF–ChIPr
GB–ChIPr
Random
DNN–ChIPr
RF–ChIPr
GB–ChIPr
Random
DNN–ChIPr
RF–ChIPr
GB–ChIPr

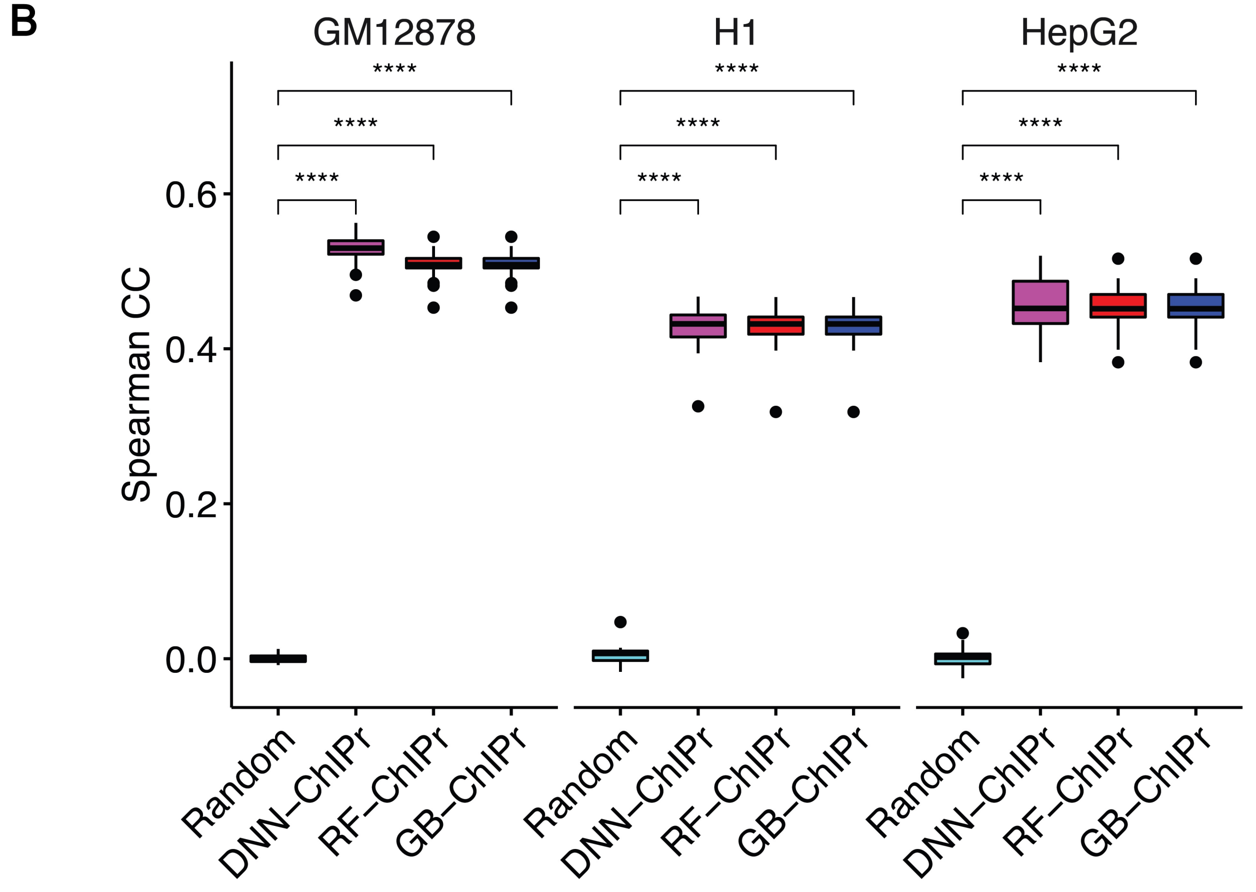
B
GM12878
H1
HepG2
****
****
****
****
****
****
****
****
****
Spearman CC
0.6
0.4
0.2
0.0
Random
DNN–ChIPr
RF–ChIPr
GB–ChIPr
Random
DNN–ChIPr
RF–ChIPr
GB–ChIPr
Random
DNN–ChIPr
RF–ChIPr
GB–ChIPr

Fig. S2

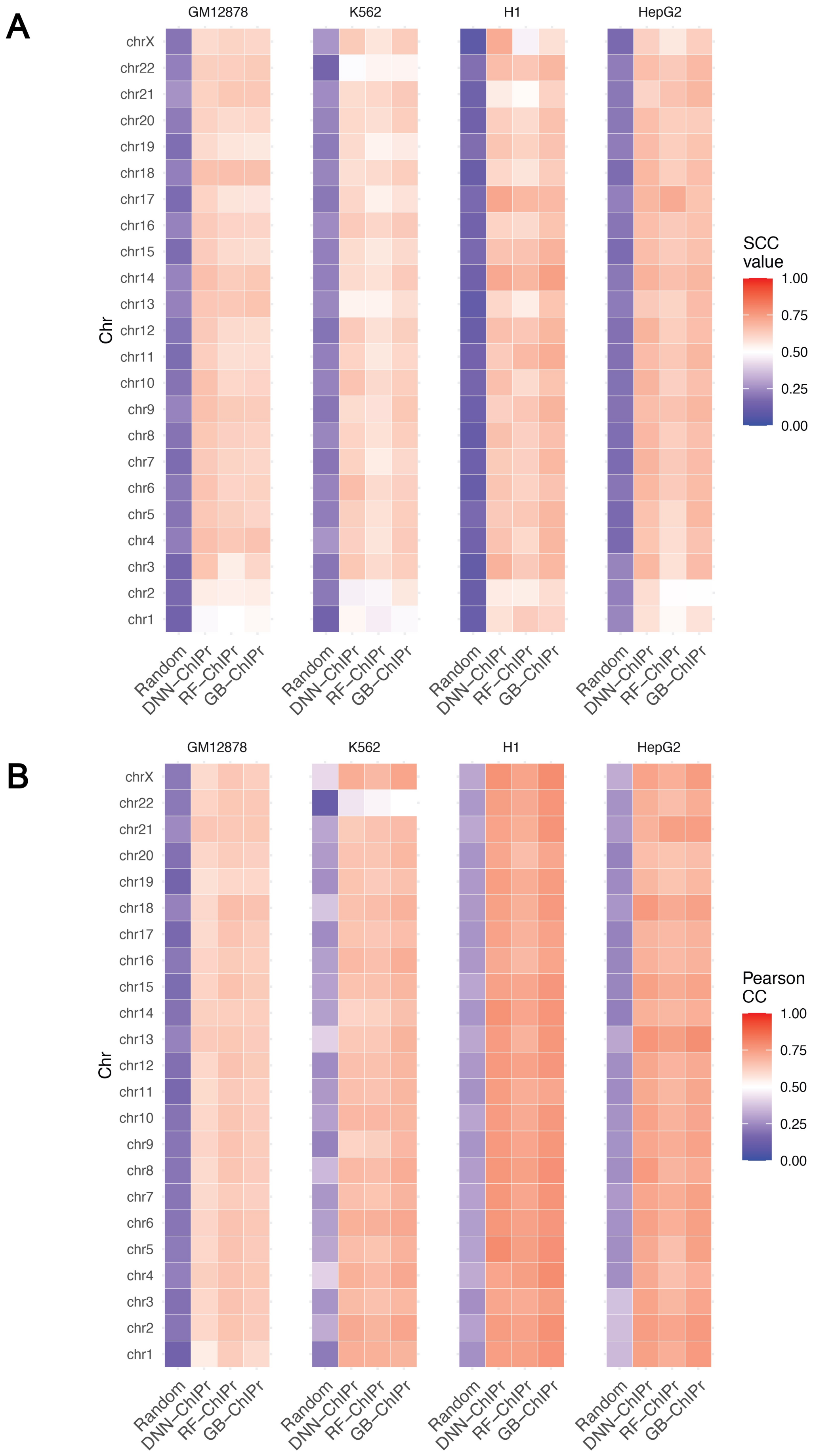
A
GM12878
K562
H1
HepG2
Chr
chrX
chr22
chr21
chr20
chr19
chr18
chr17
chr16
chr15
chr14
chr13
chr12
chr11
chr10
chr9
chr8
chr7
chr6
chr5
chr4
chr3
chr2
chr1
Random
DNN–ChIPr
RF–ChIPr
GB–ChIPr
SCC value
1.00
0.75
0.50
0.25
0.00
B
Pearson CC

Fig. S3

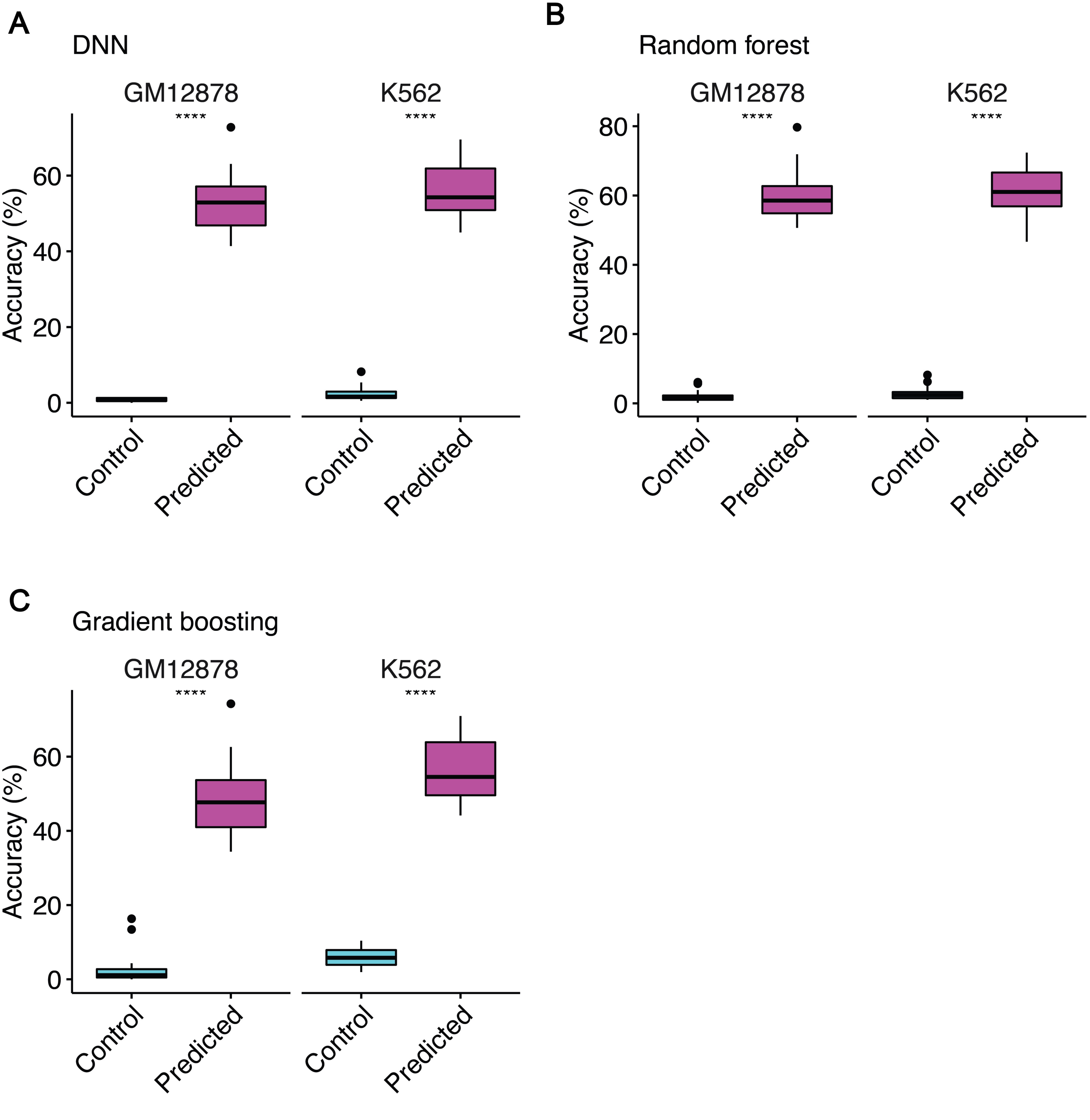

A
DNN
GM12878
K562
****
****
Accuracy (%)
0
20
40
60
Control
Predicted
Control
Predicted
B
Random forest
GM12878
K562
****
****
Accuracy (%)
0
20
40
60
80
Control
Predicted
Control
Predicted
C
Gradient boosting
GM12878
K562
****
****
Accuracy (%)
0
20
40
60
Control
Predicted
Control
Predicted

Fig. S4

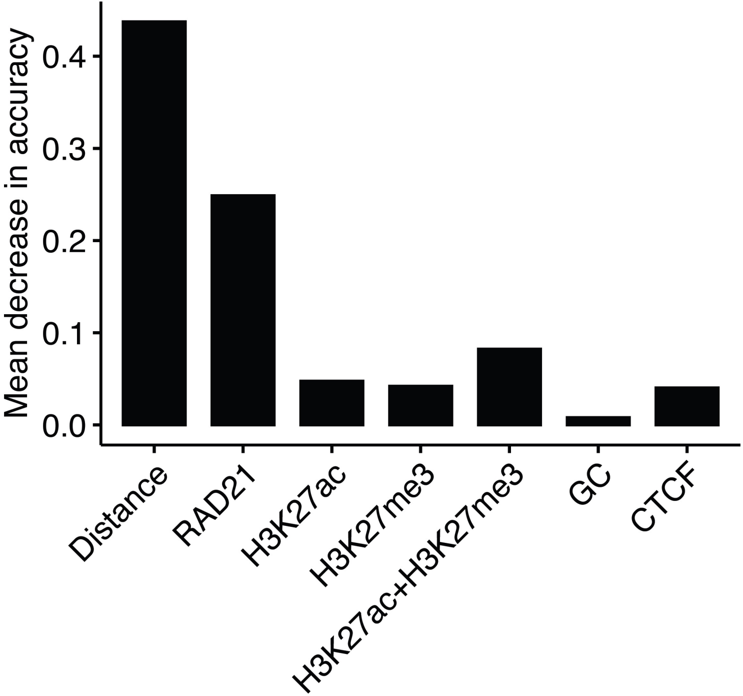
Mean decrease in accuracy
0.4
0.3
0.2
0.1
0.0
Distance
RAD21
H3K27ac
H3K27me3
H3K27ac+H3K27me3
GC
CTCF

Fig. S5

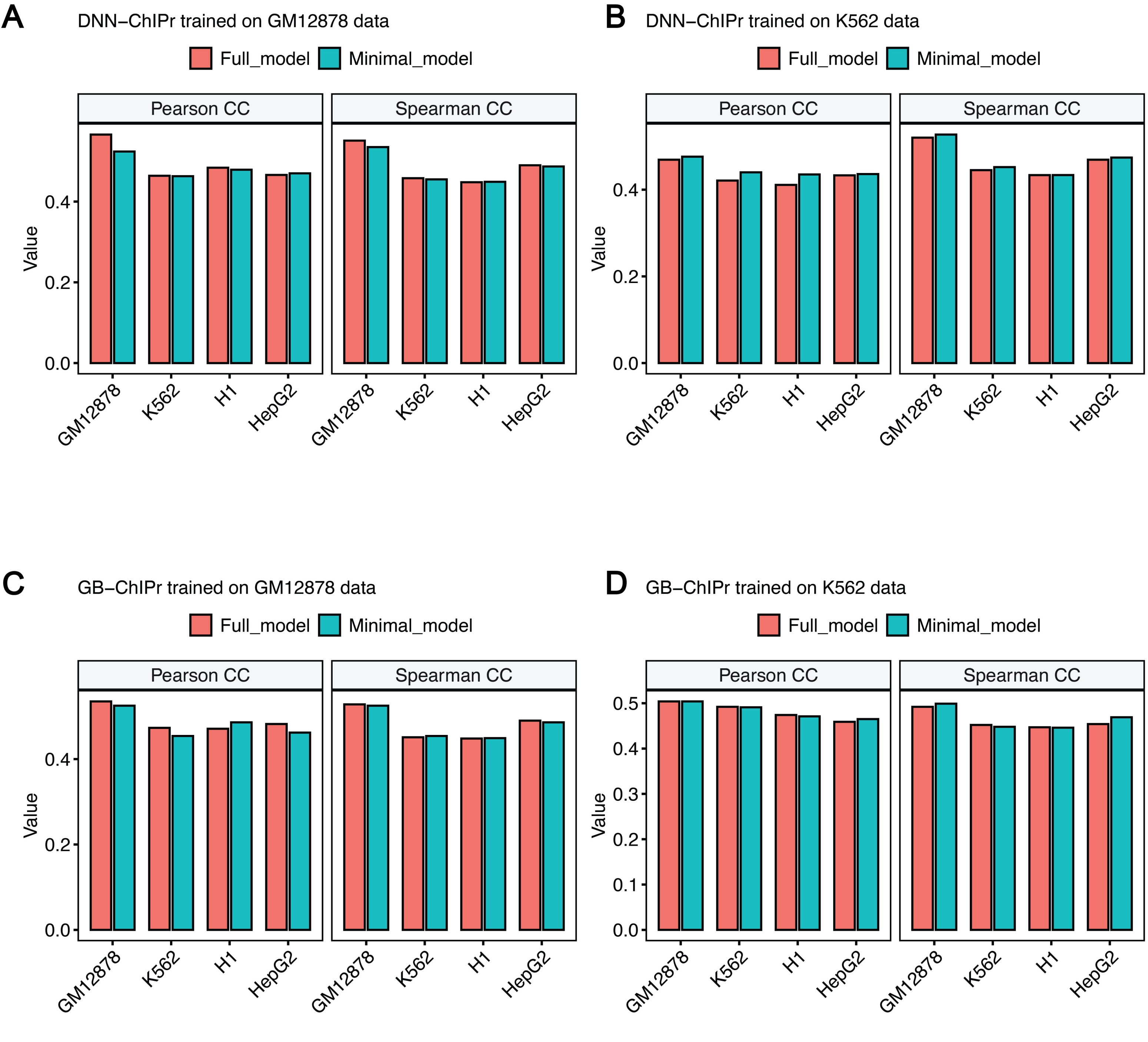
A
DNN−ChIPr trained on GM12878 data
Full_model
Minimal_model
Pearson CC
Spearman CC
Value
0.0
0.2
0.4
GM12878
K562
H1
HepG2
B
DNN−ChIPr trained on K562 data
C
GB−ChIPr trained on GM12878 data
D
GB−ChIPr trained on K562 data
0.1
0.3
0.5

Fig. S6

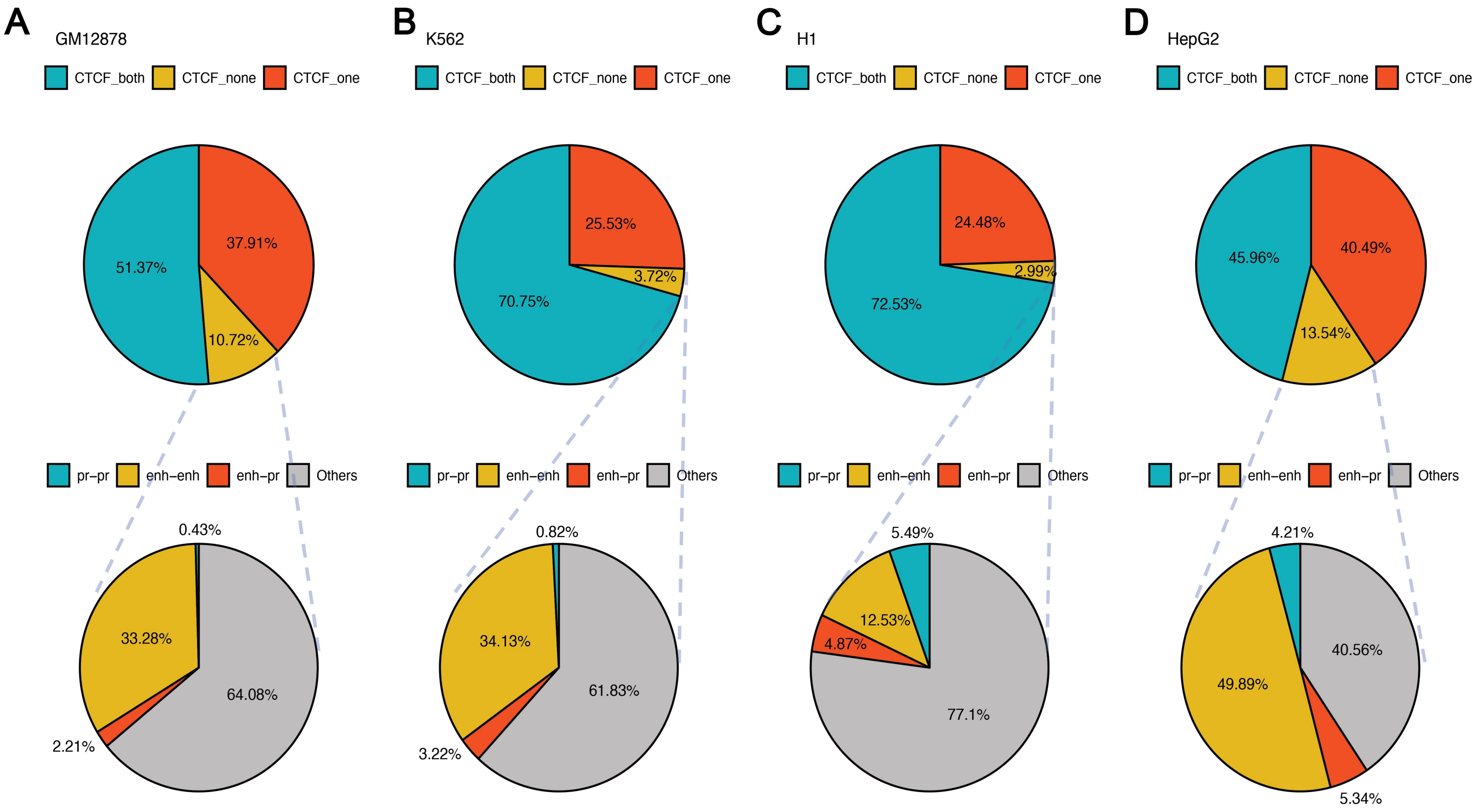

A
GM12878
CTCF_both
CTCF_none
CTCF_one
51.37%
37.91%
10.72%
pr−pr
enh−enh
enh−pr
Others
0.43%
33.28%
64.08%
2.21%
B
K562
CTCF_both
CTCF_none
CTCF_one
25.53%
3.72%
70.75%
pr−pr
enh−enh
enh−pr
Others
0.82%
34.13%
61.83%
3.22%
C
H1
CTCF_both
CTCF_none
CTCF_one
24.48%
2.99%
72.53%
pr−pr
enh−enh
enh−pr
Others
5.49%
12.53%
4.87%
77.1%
D
HepG2
CTCF_both
CTCF_none
CTCF_one
45.96%
40.49%
13.54%
pr−pr
enh−enh
enh−pr
Others
4.21%
40.56%
49.89%
5.34%

Fig. S7

H1 cell line

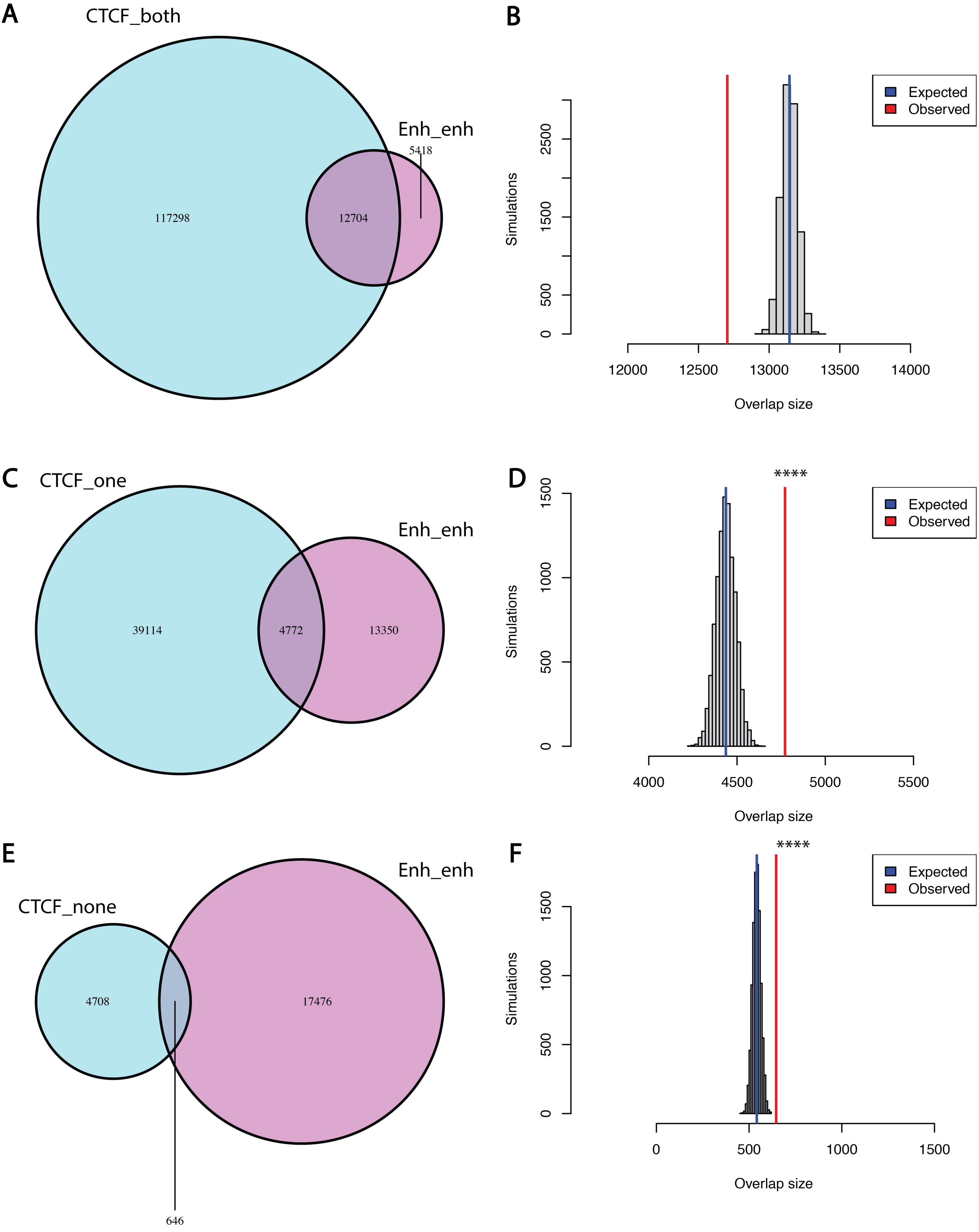

Fig. S8

HepG2 cell line

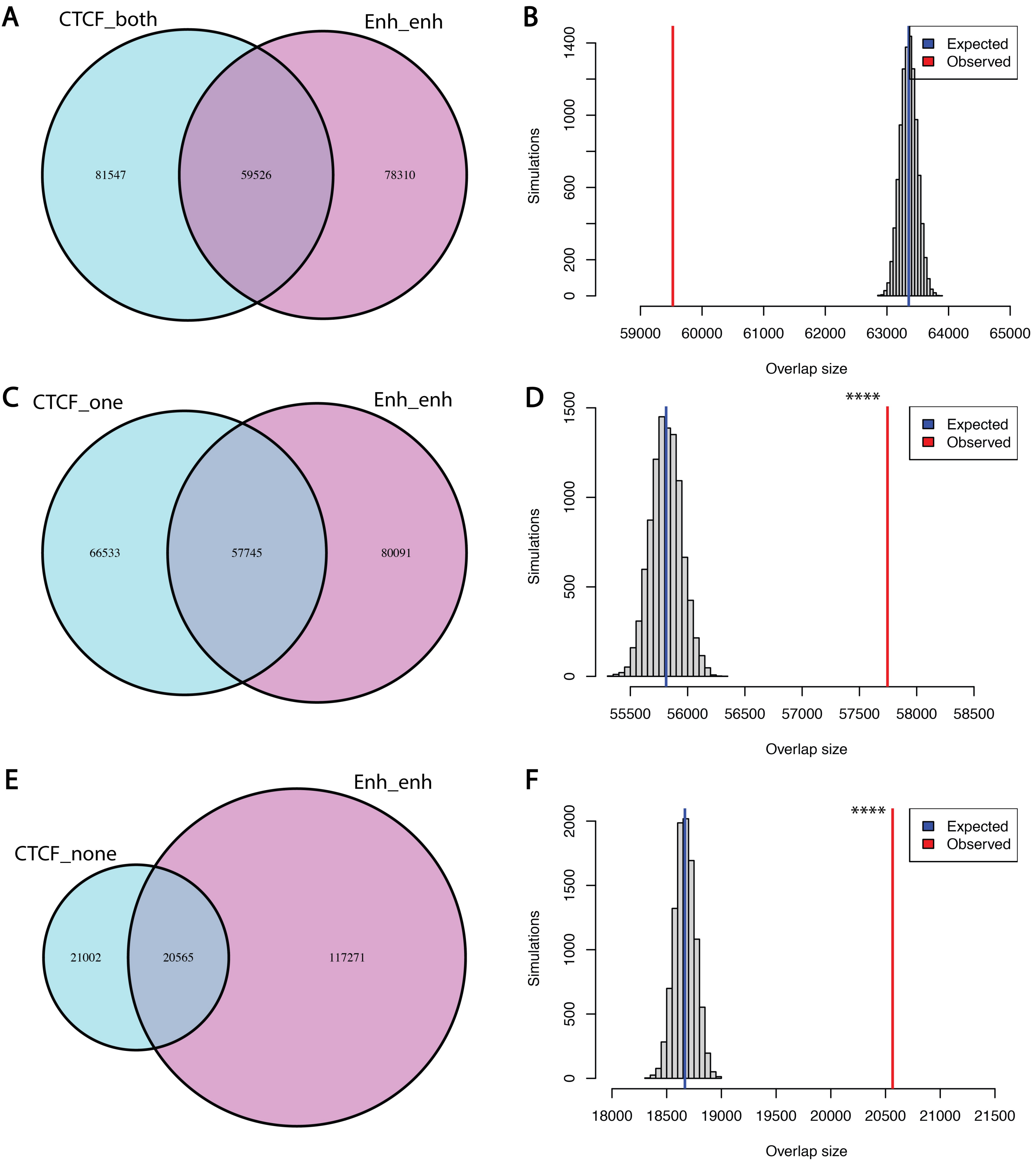

Fig. S9

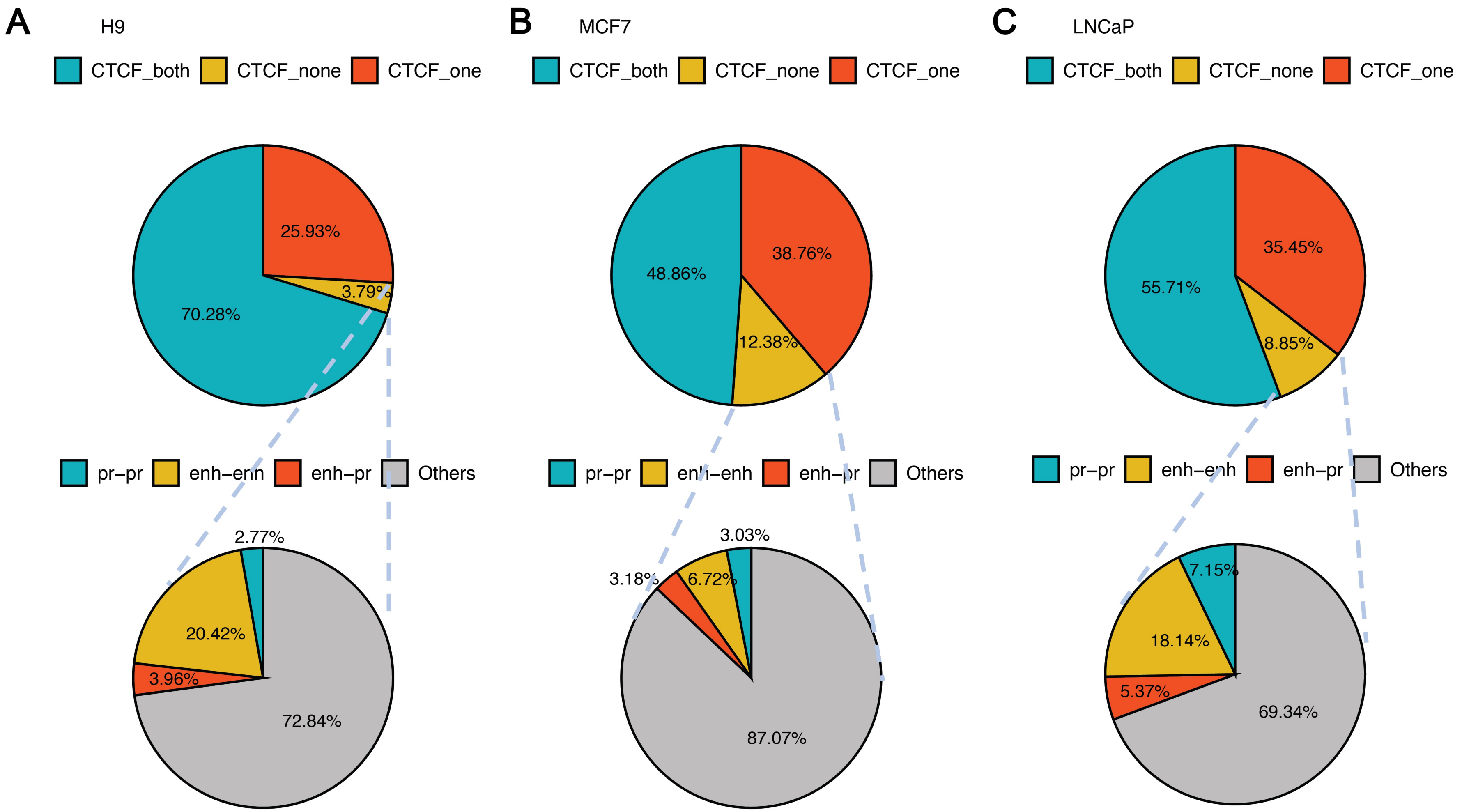

A
H9
CTCF_both
CTCF_none
CTCF_one
25.93%
3.79%
70.28%
pr–pr
enh–enh
enh–pr
Others
2.77%
20.42%
3.96%
72.84%
B
MCF7
CTCF_both
CTCF_none
CTCF_one
38.76%
48.86%
12.38%
pr–pr
enh–enh
enh–pr
Others
3.03%
3.18%
6.72%
87.07%
C
LNCaP
CTCF_both
CTCF_none
CTCF_one
35.45%
55.71%
8.85%
pr–pr
enh–enh
enh–pr
Others
7.15%
18.14%
5.37%
69.34%

Fig. S10
H9 cell line

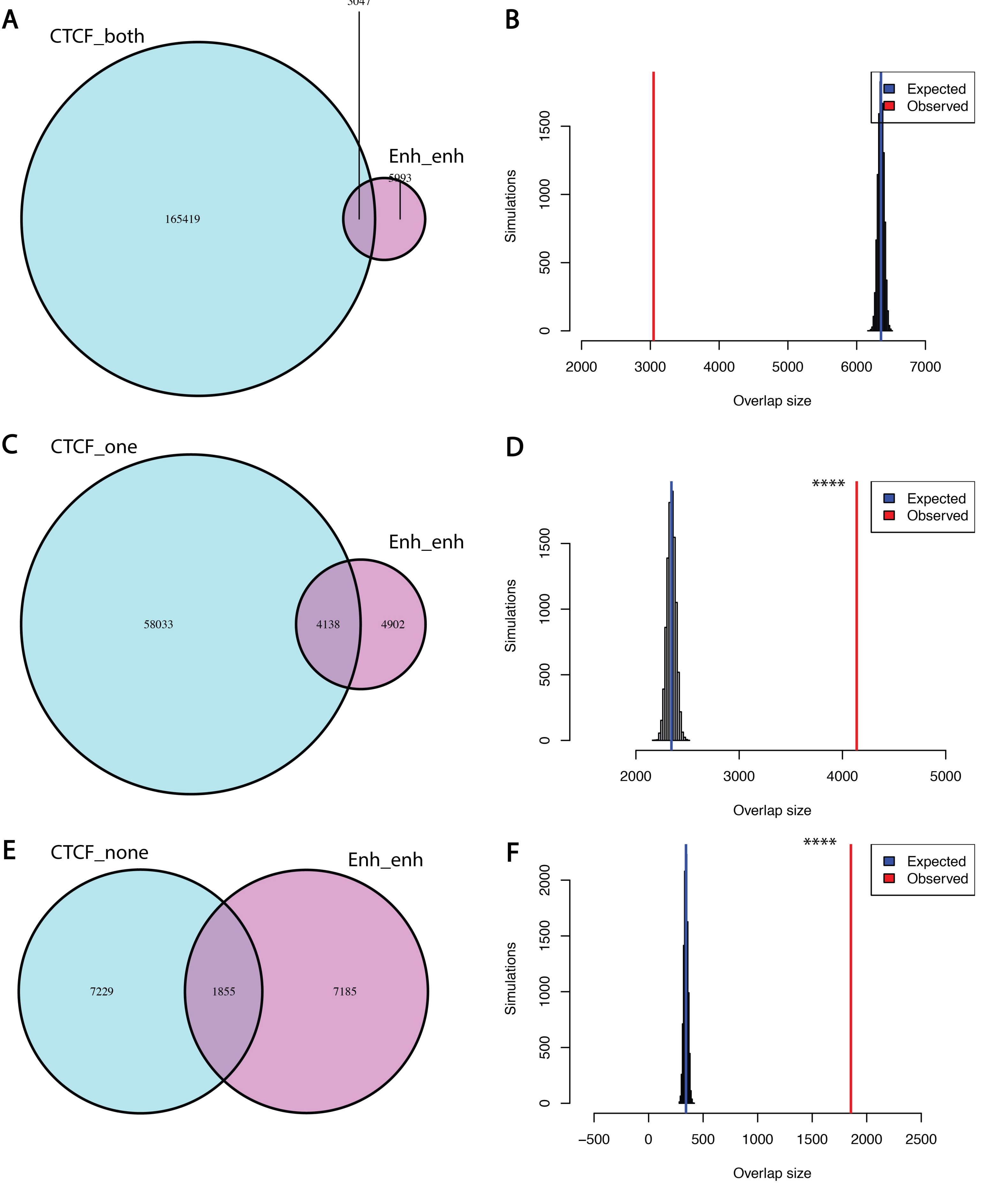

Fig. S11

MCF7 cell line

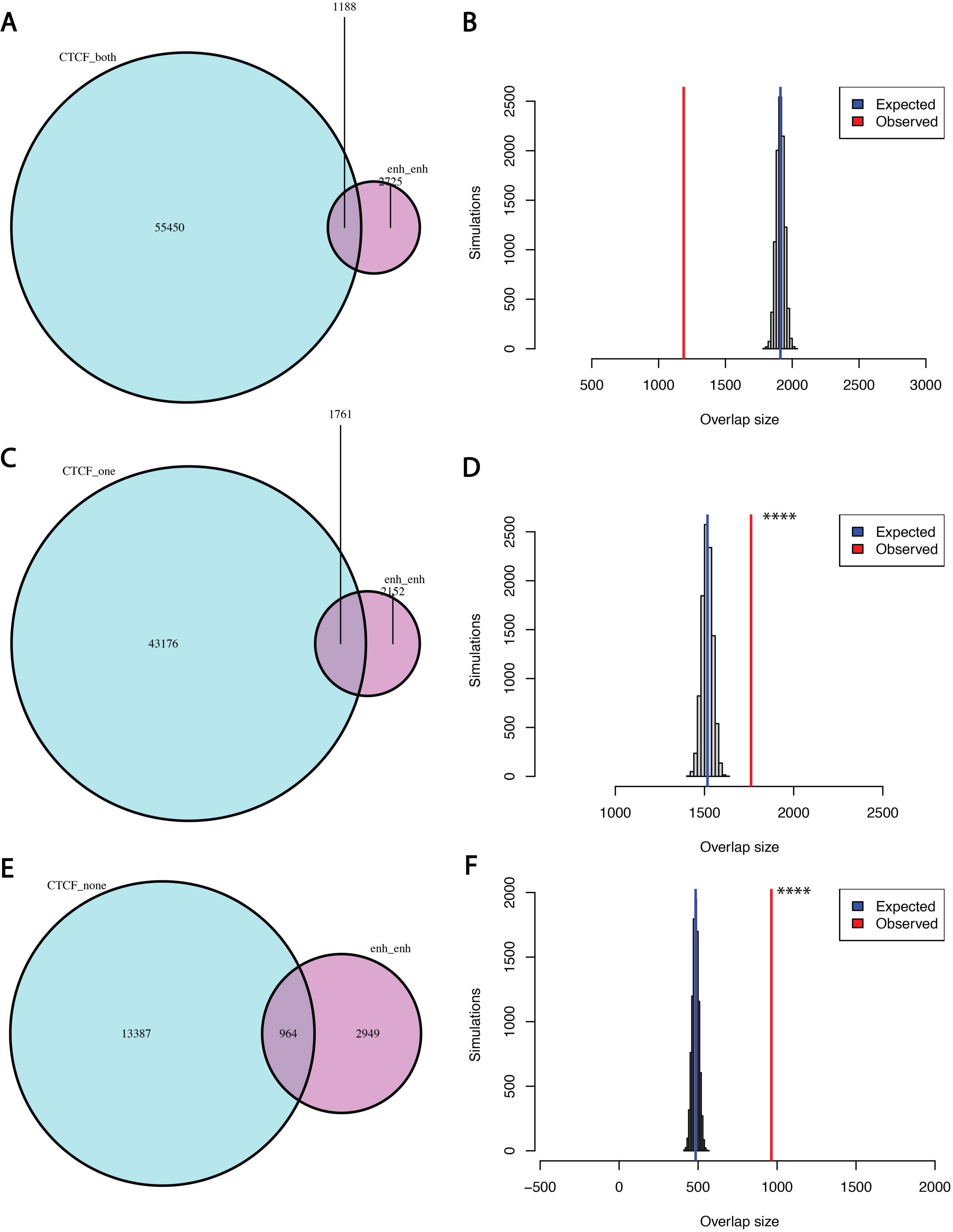

Fig. S12

LNCaP cell line

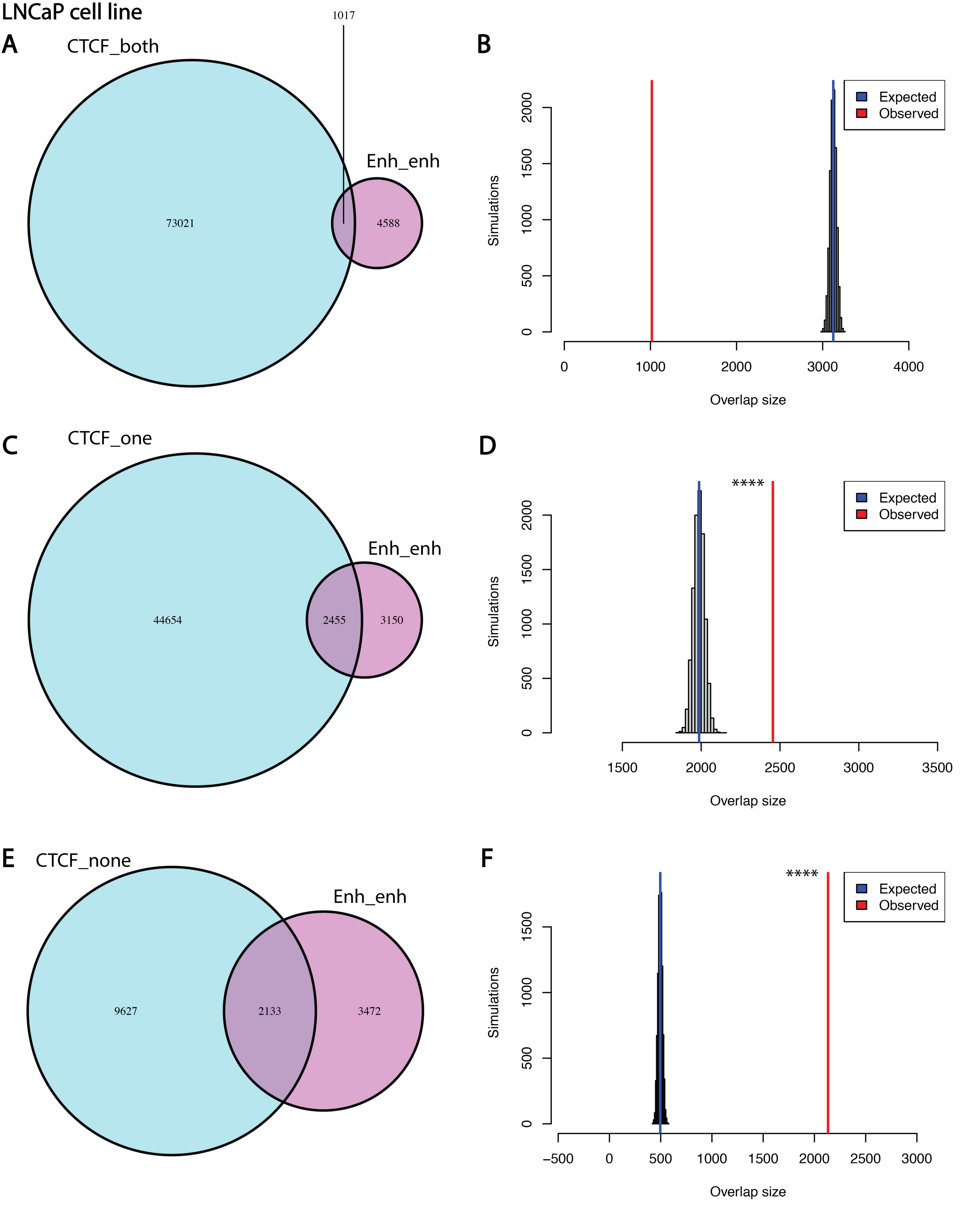

Fig. S13

BORIS(Zf)/K562-CTCFL-ChIP-Seq(GSE32465)/Homer

Match Rank: 1
Score: 0.91
Offset: -1
Orientation: reverse strand
Alignment: -CCACTAGRGGGC-------
GCCASCAGGGGGCGCYVNNG

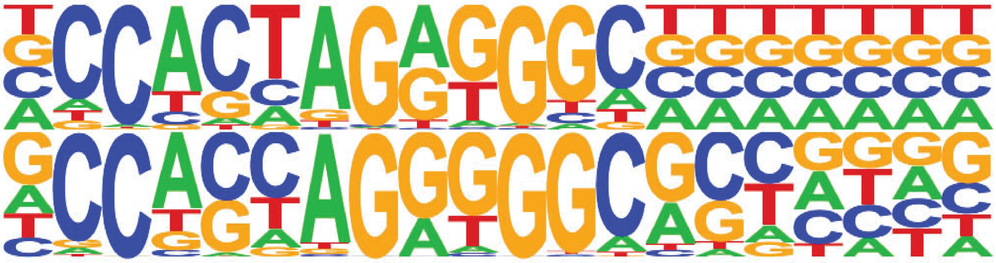

CTCF/MA0139.1/Jaspar

Match Rank: 2
Score: 0.90
Offset: -3
Orientation: forward strand
Alignment: ---CCACTAGRGGGC----
TGGCCACCAGGGGGCGCTA

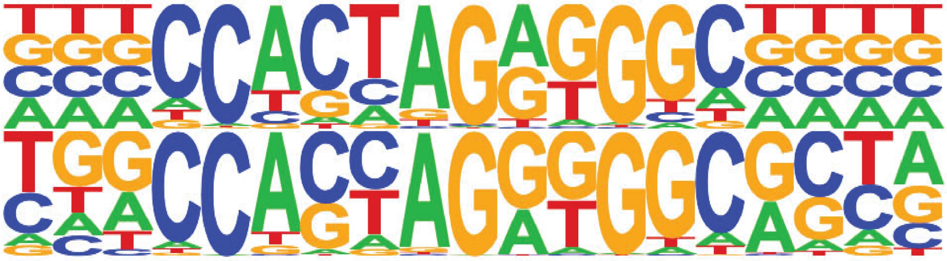

CTCF(Zf)/CD4+-CTCF-ChIP-Seq(Barski_et_al.)/Homer

Match Rank: 3
Score: 0.90
Offset: -3
Orientation: reverse strand
Alignment: ---CCACTAGRGGGC-----
TGGCCACCAGGTGGCACTNT

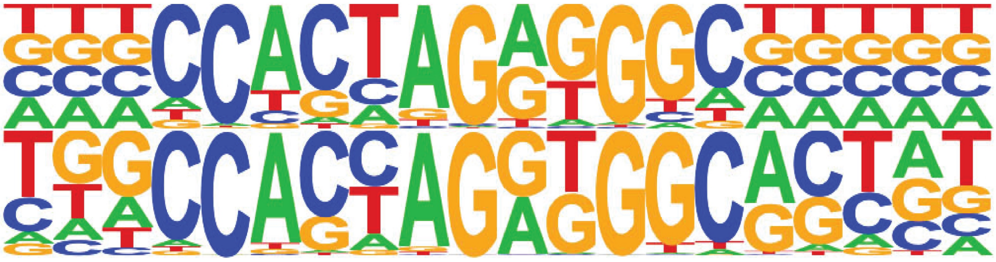

PB0076.1_Sp4_1/Jaspar

Match Rank: 4
Score: 0.60
Offset: 1
Orientation: reverse strand
Alignment: CCACTAGRGGGC------
-NNNAAGGGGGCGGGNNN

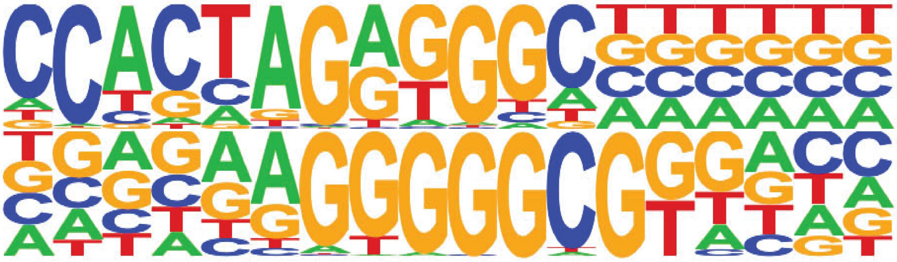

Fig. S14

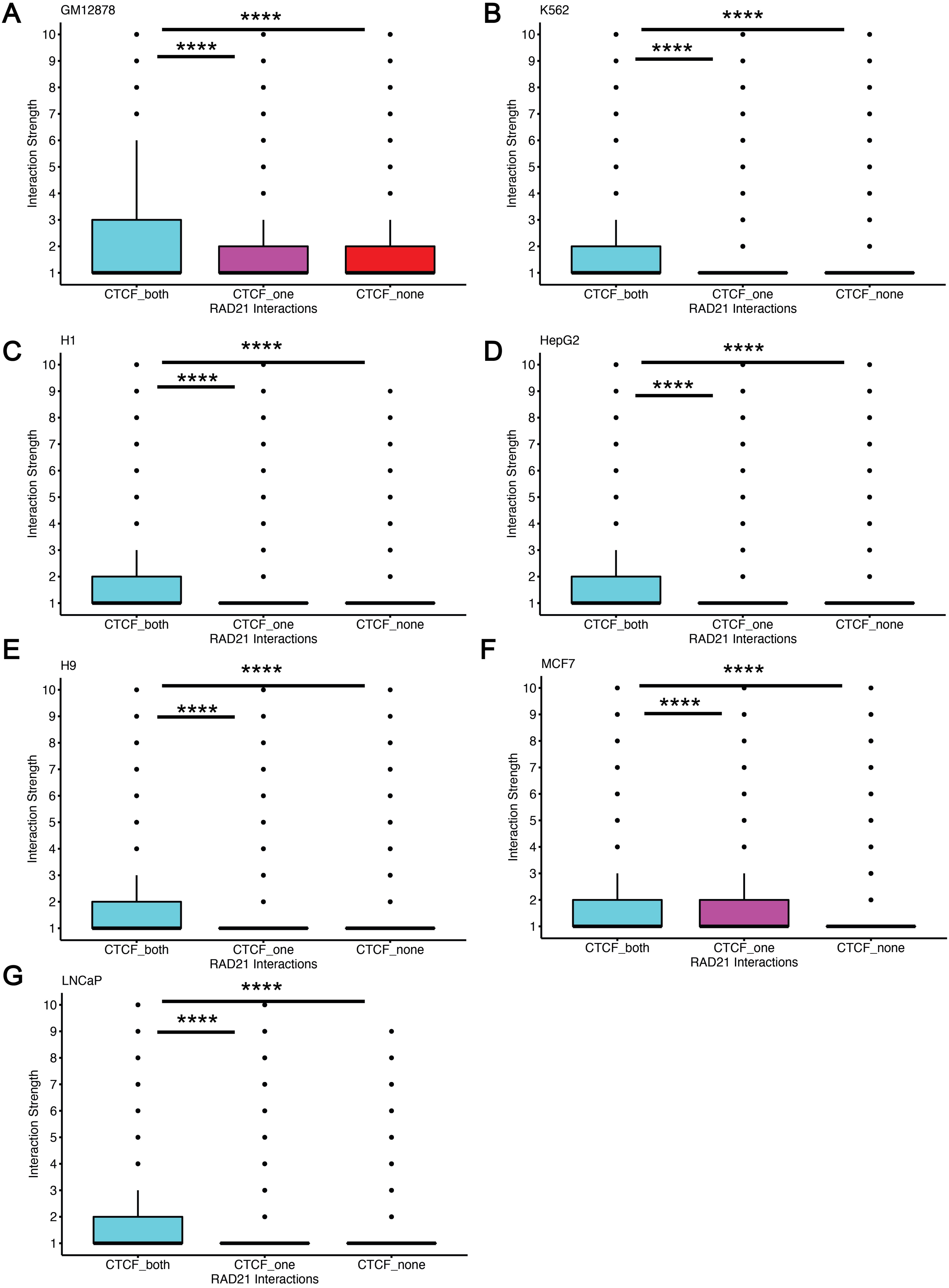
A
GM12878
B
K562
C
H1
D
HepG2
E
H9
F
MCF7
G
LNCaP
****
Interaction Strength
1
2
3
4
5
6
7
8
9
10
CTCF_both
CTCF_one
CTCF_none
RAD21 Interactions
