# Supplementary material for: ChIPr: accurate prediction of cohesin-mediated 3D genome organization from 2D chromatin features
Source: Genome Biol. 2024 Jan 12;25:15. doi: 10.1186/s13059-023-03158-7 (PMC10785520; doi:10.1186/s13059-023-03158-7)
Supplement: Supplementary file 5 — Additional file 5. Review history. [file 13059_2023_3158_MOESM5_ESM.docx]

**1^st^ round**

**Reviewer 1**

In this manuscript, Abbas et al. developed Chromatin Interaction Predictor (ChIPr), a suite of regression models based on deep neural networks, random forest, and gradient boosting, respectively, to predict cohesin-mediated chromatin interaction strength. Both standard and minimal ChIPr models can accurately capture most cell type-dependent loops identified by ChIA-PET and Hi-C. Furthermore, the authors demonstrated that with a single experimental data (Rad21 ChIP-seq), ChIPr could predict cohesin-mediated chromatin interactions with high accuracy.

Genomic sequence, epigenetic chromatin states, and protein distribution have been used to predict 3D genome structures. Among these features, both CTCF and cohesin are two critical factors that are required for chromatin loop and TAD formation. From many datasets generated from different labs, it is well known that the binding sites of CTCF and cohesin (most of them co-localize) correlate well with TAD borders and loop anchors. In addition, CTCF and cohesin distribution in the human genome are pretty conserved in different types of cells. Therefore, it is unsurprising that the authors can successfully predict chromatin structures using Rad21 ChIP-seq datasets in other cells.

The authors proposed that ChIPr is especially useful if cells are limited and only enough for a ChIP experiment. The experimental difficulty, cost, and cell number required are not so different between Hi-C and ChIP-seq. ChIP requires antibodies, and the quality of antibodies dictates the outcome of ChIP-seq results. Unfortunately, the ChIP-grade antibody for Rad21 has not been commercially available in recent several months. Then, the availability of a suitable antibody could be a problem for a ChIP experiment. If a limited number of cells are available, I would suggest doing Hi-C instead of Rad21 ChIP-seq, considering many potential uncertainties.

This manuscript is well written, well presented, and easy to read and understand. However, I am afraid the method itself may not find broad utility or interest to others in the field or a broad audience of biologists. Nevertheless, I believe some biologists and some bioinformaticians may be interested in using ChIPr.

**Reviewer 2**

In their paper "Accurate prediction of cohesin-mediated 3D genome organization from 2D chromatin features", Abbas et al. present a computational tool for predicting ChIA-PET based interactions using three ChIP-seq data sets. The authors explore 3 different machine learning methods, showing that the model can generate good predictions for several cell types.

Unfortunately I found this paper difficult to follow, with a lot of key details and explanations missing. Partriculalry, the metrics which the authors used to compare their predictions with data were not explained in enough detail for the reader to get a proper understanding of how well the method works. I think it would also be useful if the authors explored whether the scheme could be applied to other types of data. ChIA-PET is now quit an old technology, and has largely been superseded by more recent methods such as Hi-ChIP, OCEAN-C, DNase-C, etc. which tend to give higher quality data. If the model in its present for only performs well at predicting ChIA-PET data, it is unclear to me how useful this will be for readers of Genome Biology.

Finally, I note that the manuscript was submitted as a 'method' paper, and so in its self does not provide any (or very little) new biological insight. At the same time, although the authors describe ChiPr as a 'a suite of
regression models', they do not provide any detail on how a reader might use their methods. They don't provide any software or scripts, and the methods section is too vague to be used to reproduce what has been done.

For the above reasons, I cannot recommend that the paper is accepted in its present form. Below I give some further details and suggestions for where the manuscript could be improved.


Detailed comments:

Note that the pages are not numbered, and line number start again on each page, which makes commenting difficult.

1. Introduction: the authors mention three regression models, DNN, RF and GB. No explanation of the differences between these models is given, and there are no references. For the readership of Genome Biology, who may be unfamiliar with these methods, I think some explanation needs to be given.

2. Introduction: the authors state a hypothesis that there are 6 features which will control the interaction strength between any to genomic positions. Two of these are histone modifications. It would be good if some rational and background for this hypothesis was given - why these two particular modifications and not others.

3. Results: Peak-level predictions. There are a lot of details lacking here.

- how do they initially identify peaks? Is this from peak calls on Rad21 ChIP-seq?

- How is the model trained, just using peak information, or the whole ChIA-PET data set?

- One of the input data sets is CTCF motif orientation. How is this obtained - do they search for motifs only within the Rad21 ChIP peaks, is CTCF ChIP data used? How do they deal with multiple motifs appearing within a peak etc.? Is information on how well a given motif matches the consensus used?

- Other input data include histone ChIP-seq and Rad21 ChIP-seq; how are these data sets used in the model? Are peaks called, and peak positions and p-values used, or is a 'fold enrichment over input' track generated?

- In this section, to evaluate performance, the authors generated random values for the interaction strengths to use as a control. This was a bit unclear. Particularly, do they correct for the genomic distance between the peaks (they do this in some of the metrics used later)? How they calculate the correlation was also not clear. Is the data set the interaction strength for all possible pairs of peaks, and they find the correlation between prediction and data?

- It would be useful to also offer some other metrics of the quality of prediction. Fraction of correctly or incorrectly predicted interactions etc.

- There are several statements in the text where a numerical value of p-value would help. e.g. "the predicted outputs of the three different variants of ChIPr correlated significantly better with the..." could quote some numbers here. Later they say results for different cell lines as "quite similar", again some quantification would be useful.

4. Results: ChIA-PET at 25 and 5kbp bins

- Between this and the previous section, it is not clear what output the authors model actually gives. Is it a binned map from which they then call peaks, or is it a set of peaks from which they then generate a binned map. Or are these two separately trained models?

- In this section they use a weighted sum of Pearson correlation coefficients from different distance strata as a quality metric. More detail of this calculation would be useful. For example, a 'smoothing half size' is mentioned, but not explained in the main text. A diagram of some sort might help.

- There is a statement that predicted maps correlate significantly with the original maps. Again, some numbers in the text would be helpful.

5. Results: ChIPr captures both cell-type dependent and universal...

- The authors state that using all three ChIPr models would give the most robust view. This is not well explained - should results from the 3 models be combined in some way, or just considered side by side?

6. Results: Contributions of input features to the ChIPr predictions

- The authors talk about a 'mean absolute error value', but don't explain what this means.

- Again in this section some numbers in the text could be useful. The authors talk about 'a small difference' between cases where they do not include one of the ChIP-seq data sets and a 'bigger drop' when they do not include two data sets.

7. Results: minimal model section and CTCF section

- It is unclear whether authors are considering peak-level or binned interaction map level data here (or both)

- Again, how the authors obtain CTCF motif information is unclear. Do they search the entire genome for CTCF motifs, or only Rad21 peaks? When they identify CTCF motifs, to they consider a score of how closely the motif matches the consensus motif?

**Authors’ response**

*Reviewer 1:*

*C1: In this manuscript, Abbas et al. developed Chromatin Interaction Predictor (ChIPr), a suite of  regression models based on deep neural networks, random forest, and gradient boosting, respectively, to  predict cohesin-mediated chromatin interaction strength. Both standard and minimal ChIPr models can  accurately capture most cell type-dependent loops identified by ChIA-PET and Hi-C. Furthermore, the  authors demonstrated that with a single experimental data (Rad21 ChIP-seq), ChIPr could predict  cohesin-mediated chromatin interactions with high accuracy.*

**Response:** Thank you for the positive summary.

*C2: Genomic sequence, epigenetic chromatin states, and protein distribution have been used to predict  3D genome structures. Among these features, both CTCF and cohesin are two critical factors that are  required for chromatin loop and TAD formation. From many datasets generated from different labs, it is  well known that the binding sites of CTCF and cohesin (most of them co-localize) correlate well with TAD  borders and loop anchors. In addition, CTCF and cohesin distribution in the human genome are pretty  conserved in different types of cells. Therefore, it is unsurprising that the authors can successfully predict  chromatin structures using Rad21 ChIP-seq datasets in other cells.*

**Response:** Thank you for pointing this out. Although, CTCF and cohesin distribution in the human  genome is well conserved in different types of cells, it is increasingly clear that there are also cell-type  specific features/loops of CTCF and RAD21 (in addition to the universal features). This is nicely  summarized in the Grubert et. al. study (Nature. 2020 PMID: 32728247).

The main assumption in ChIPr is that chromatin interaction strength between anchor loci in a given cell  line depends on some epigenomic features of the same cell line. Through training ChIPr, it tries to learn  the implicit relation between these input epigenomic features and the output interaction strength for a  given cell line. We assume that this relation learned by the model holds for any cell line. Different cell

lines will have different transcription patterns according to the different chromatin states they have  including histone modifications (Karlić et. al. Proc. Natl. Acad. Sci. 2010 PMID: 20133639). To  predict the interaction strengths of another cell line (other than the one used in training the model), we  use the same model with the epigenetic features of this new cell line. As shown in Fig. 6 in the  manuscript, the output interactions predicted differ from one cell line to another according to the input  features for each cell line. For instance, the area upstream of the *MYC gene* has a lot of enhancers in  GM12878 and very few enhancers in K562. In line with this, we see a lot of predicted strong interactions  in GM12878 and much fewer interactions in K562. Similarly, we find the interaction pattern is different  in the other two cell lines H1 and HepG2. In addition, we see a lot of strong predicted interactions  between MYC and its downstream enhancers in K562 (a cancer cell line). We do not see a similar pattern  in the other three cell lines which are normal.

In summary, we conclude that ChIPr can accurately predict both cell-type specific and universal  cohesion-mediated chromatin interactions.

*C3: The authors proposed that ChIPr is especially useful if cells are limited and only enough for a ChIP  experiment. The experimental difficulty, cost, and cell number required are not so different between Hi-C  and ChIP-seq. ChIP requires antibodies, and the quality of antibodies dictates the outcome of ChIP-seq  results. Unfortunately, the ChIP-grade antibody for Rad21 has not been commercially available in recent  several months. Then, the availability of a suitable antibody could be a problem for a ChIP experiment. If  a limited number of cells are available, I would suggest doing Hi-C instead of Rad21 ChIP-seq, considering  many potential uncertainties.*

**Response:** Thank you for this important comment.

The ENCODE portal reported high-quality RAD21 ChIA-PET datasets from 24 cell lines using the Anti Rad21 rabbit polyclonal antibody (ab992) from Abcam (Grubert et. al. Nature. 2020 PMID: 32728247).  Independently, we tested the same RAD21 antibody (cat: ab992) and were able to generate high quality  ChIA-PET datasets from a panel of prostate cancer and benign prostate cell lines (these datasets are  intended for a future publication). Although the company Abcam does not explicitly certify the Anti Rad21 antibody (ab992) as ChIP-grade, we and others have obtained excellent ChIP and ChIA-PET data  from this antibody. The significant overlap of RAD21 peaks with CTCF peaks (and CTCF motif) attests to  the high quality of these data. Furthermore, the recombinant Anti-Rad21 antibody [EPR22506-15] from  Abcam is also ChIP-grade. But we have not (yet) used this antibody in our laboratory. Both these  antibodies (ab992 and EPR22506-15) are currently available in the Abcam catalogue. In the past, Abcam  listed the Anti-Rad21 rabbit polyclonal antibody (ab992) as ChIP grade, but no longer call this antibody  ChIP grade. We contacted the company for the reason for removing the ChIP grade designation. Abcam  informed us that “they decided to remove the ChIP application as they are not currently performing  quality control testing in ChIP for every new batch of this product. This doesn't mean, however, that the  product is not suitable for this application, it just means it is not tested every time a new batch is  produced.” In summary, based on our investigation, we do not consider the antibody related concern to  be a significant concern. We suggest that supply-chain issues regarding the availability of Rad21  antibody is a short-term problem. Rather, our study will stimulate more vendors to make better Rad21  antibodies (which is important for the field).

Hi-C is a powerful tool to identify topologically associating domains (TADs). However, in general, Hi-C  studies have limited utility in the precision mapping of enhancer-promoter contacts. We stated this  problem with Hi-C in the introduction section of our paper in JCI (Ramanand et. al. J Clin Invest. 2020  PMID: 32343676). Other groups have also highlighted the limitation of Hi-C in deciphering the fine-scale  enhancer connectome (Tsung-Han S Hsieh et. al. Mol Cell. 2020 PMID: 32213323). If the goal is to  identify (lower resolution) TADs, then Hi-C maybe suitable. However, if the goal is to obtain higher  resolution enhancer-promoter contacts, our ChIPr method is likely to be better suited. Our ChIPr  method will empower the scientific community to identify cell-type specific and high-resolution  chromatin contact maps.

Despite these limitations, we do agree that Hi-C experiments with extreme sequencing depth (>1 billion  reads) can provide enhancer-promoter contacts as described in the Rao et. al. study (Rao et. al. Cell.  2014 PMID: 25497547). Such a high sequencing depth will cost several thousands of dollars. A cohesin  ChIP-Seq experiment typically requires about 30 million reads. While we agree that the cost of a ChIP Seq with 30 million reads will be comparable to the cost of Hi-C with equal number of reads (costing

about a few hundred dollars), the real utility of ChIPr is in identifying enhancer-promoter contacts with a  simple ChIP-Seq experiment.

In addition, there are many cell lines with currently available RAD21 ChIP-seq data and no ChIA-PET  data. We can apply ChIPr on these datasets to predict cohesion-mediated chromatin interactions in the  corresponding cell lines. It is also worth noting that ChIPr is a simple, straightforward method. It can be  used (by adding or modifying some input features) to predict CTCF and RNA-POL II-associated  interactions’ strengths. Going forward, we anticipate that ChIPr may have utility in identifying chromatin  interactions in patient biopsies/specimens.

*C4: This manuscript is well written, well presented, and easy to read and understand. However, I am  afraid the method itself may not find broad utility or interest to others in the field or a broad audience of  biologists. Nevertheless, I believe some biologists and some bioinformaticians may be interested in using  ChIPr.*

**Response:** Thank you for pointing this out. We have made the source code of ChIPr publicly available at  https://git.biohpc.swmed.edu/s206442/chipr. A detailed Readme file is available for training the  regression models using the data of a representative cell line (GM12878) and testing it on the data of  another cell line (K562). The users can test or use the model to predict the interaction strength of any  cell line provided that its ChIP-seq data is available by just giving the links of the ChIP-seq data in the  code (in specific lines described in the Readme file). Currently, ChIPr supports input files generated using  the hg19 reference genome only. However, users can use liftover [https://genome.ucsc.edu/cgi bin/hgLiftOver] in case the files were generated using hg38. Also, the code is commented, making it easy  for developers to use the code and modify it according to their needs.

**Reviewer #2:**

*C1: In their paper "Accurate prediction of cohesin-mediated 3D genome organization from 2D chromatin  features", Abbas et al. present a computational tool for predicting ChIA-PET based interactions using  three ChIP-seq data sets. The authors explore 3 different machine learning methods, showing that the  model can generate good predictions for several cell types.*

**Response:** Thank you for the nice summary.

*C2: Unfortunately I found this paper difficult to follow, with a lot of key details and explanations missing.  Partriculalry, the metrics which the authors used to compare their predictions with data were not  explained in enough detail for the reader to get a proper understanding of how well the method works. I  think it would also be useful if the authors explored whether the scheme could be applied to other types  of data. ChIA-PET is now quit an old technology, and has largely been superseded by more recent  methods such as Hi-ChIP, OCEAN-C, DNase-C, etc. which tend to give higher quality data. If the model in  its present for only performs well at predicting ChIA-PET data, it is unclear to me how useful this will be  for readers of Genome Biology.*

**Response:** Thank you for pointing this out. We updated the paper and addressed all your detailed  comments below to make the paper clearer and easier to understand. In principle, ChIPr is a very  straightforward method and it can be used with other data types. We can add more ChIP-seq inputs if  needed and we can use inputs of fixed-size bins instead of inputs at the peak-level resolution. All we  need to do is to train the regression models with the desired outputs of the other data type rather than  the ChIA-PET RAD21 interactions’ strengths.

We have trained RF-ChIPr on the odd chromosomes of CTCF in-situ ChIA-PET data of the GM12878 cell  line [https://www.encodeproject.org/experiments/ENCSR184YZV/]. We have tested the model on the  even chromosomes of the same sample. We observed high Pearson and Spearman correlation values at  the peak-level resolution (in the same range as the values we obtained for cohesin-mediated  interactions) (Fig. 1). These results, alongside the simple design of the model, show that ChIPr’s utility  can be extended to other proteins in addition to RAD21.

As for ChIA-PET data, we believe it is still very useful. ChIA-PET captures interactions mediated by a  specific protein of interest. It has recently been used to determine the landscape of cohesin-mediated  chromatin loops in the human genome (Grubert, Srivas et al. 2020). In addition, it has also been used to  determine the landscape of RNA polymerase II-associated chromatin interactions in prostate cancer  (Ramanand, Chen et al. 2020). For this study, we focused on ChIA-PET data prediction because of the  availability of several high-quality datasets. We agree with the reviewer that there are additional  excellent methods such as Hi-ChIP, OCEAN-C, DNase-C, among others. But a comprehensive analysis of  all these datasets is beyond the scope of this paper. Our major goal was to predict cohesion mediated  chromatin interaction and that’s the reason for choosing the gold-standard cohesin ChIA-PET datasets from ENCODE.

*C3: Finally, I note that the manuscript was submitted as a 'method' paper, and so in its self does not  provide any (or very little) new biological insight. At the same time, although the authors describe ChiPr  as a 'a suite of regression models', they do not provide any detail on how a reader might use their  methods. They don't provide any software or scripts, and the methods section is too vague to be used to  reproduce what has been done.*

**Response:** Thank you for pointing this out. ChIPr software mainly focuses on predicting the chromatin  interaction strength of cohesin-mediated interactions. We initially hypothesized that RAD21, H3K27ac,  and H3K27me3 ChIP-seq data alongside three easily available inputs (genomic distance, GC content, and  CTCF orientation) are suitable input features for our prediction task. Getting the results using ChIPr full  model with all inputs, we realized that ChIPr could do a good prediction job using only one ChIP-seq  experiment (RAD21) alongside the other easily available inputs (see manuscript Fig. 7A, C, and D). We  then proposed a minimal model and obtained very close results to the full model (see manuscript Fig. 7E  and F, and Fig. 8). In the future, this base model can also be used to predict the interaction strength for  master transcription factors.

In addition, we recommend using all the ChIPr variants (DNN, random forest, and gradient boosting) side  by side to accurately confirm strong or weak interaction strength predictions. If all three regression  methods of ChIPr predict strong interaction (interaction strength >= 3) between two anchor peaks, we  expect that the interaction between these two peaks is truly strong. Similarly, if the three of them  predict weak interactions (interaction strength < 3), we expect that the interaction is truly weak.  However, in some cases, the three methods do not agree in predicting certain interactions. In these  cases, we consider the interaction weak or will not fully trust the predictions.

We also have made the source code of ChIPr publicly available at

[https://git.biohpc.swmed.edu/s206442/chipr]. We have also added a detailed Readme file for training  the regression models using the data of some cell line (GM12878) and testing it on the data of another  cell line (K562). The users can test or use the model to predict the interaction strength of any cell line  provided that its ChIP-seq data is available by just giving the links of the ChIP-seq data in the code (in

specific lines described in the Readme file). Also, the code is commented, making it easy for developers  to use the code and modify it according to their needs.

*For the above reasons, I cannot recommend that the paper is accepted in its present form. Below I give  some further details and suggestions for where the manuscript could be improved.*

**Response:** We hope that we have satisfactorily addressed the reviewer comments. *Detailed comments:*

*Note that the pages are not numbered, and line number start again on each page, which makes  commenting difficult.*

*1. Introduction: the authors mention three regression models, DNN, RF and GB. No explanation of the  differences between these models is given, and there are no references. For the readership of Genome  Biology, who may be unfamiliar with these methods, I think some explanation needs to be given.*

**Response:** A neural network (NN), in its simplest form, is a system that consists of an input layer, one or  more hidden layers, and an output layer (Fig. 2). Each layer is connected to the following layer through  edges and each edge has a certain weight. The neural network model is trained by providing many  input/output examples. The model learns by gradually changing the edges' weights between layers until  the NN model's output is very close to the desired output given in the training examples. A nice  introduction to neural networks can be found at [https://www.mathworks.com/discovery/neural network.html].

Both random forest and gradient boosting are decision tree-based regression models (Fig. 3). In random  forest, the training data is divided into N subsamples with repetition. For each subsample of training  data, we build a decision tree to learn the relation between inputs and outputs in this subsample. Thus,  we will have an ensemble of N decision trees, and the final decision is obtained by majority voting (Fig.  4).

On the other hand, in gradient boosting, we start with a random guess for the output. Then, we  calculate the error, which is the difference between the output of the model and the actual desired  output. We then keep growing decision trees to the model to minimize this calculated error (Fig. 5).

In the revised introduction, we have provided a good background to DNN, RF, and GB. In addition, we  have also cited relevant articles and books (for example: (Bishop and Nasrabadi 2006), (Breiman 2001),  and (Wade 2020)).

*2. Introduction: the authors state a hypothesis that there are 6 features which will control the interaction  strength between any to genomic positions. Two of these are histone modifications. It would be good if  some rational and background for this hypothesis was given - why these two particular modifications  and not others.*

**Response:** Thank you for the comment. We used two histone modifications, H3K27ac and H3K27me3  because they correlate positively with active chromatin and inactive chromatin, respectively. We sought  that the activity of chromatin, that can be estimated by these histone marks, can be a good predictor of  the interaction strength. Although there are other histone modifications that also correlate with active

or inactive chromatin, we chose these two particular ones mainly because they are available for most of  the cell lines on the ENCODE portal. We clarified this part in subsection “Structure of ChIPr” in the  “Material and Methods” section.

*3. Results: Peak-level predictions. There are a lot of details lacking here.*

*- how do they initially identify peaks? Is this from peak calls on Rad21 ChIP-seq?*

**Response:** Thank you for the comment. The RAD21 ChIA-PET intrachromosomal interactions file  contains all the intrachromosomal interactions detected by the ChIA-PET experiment in a cell line. Each  interaction in the file is between two peaks whose coordinates are listed. We used these peaks and  obtained their epigenomic features as inputs to the regression models [1]. The original ChIA-PET  interaction strengths listed in the file were the desired outputs of the regression model during the  training process. This part is described in the subsection “Preparation of the training data” in the  “Material and Methods” Section.

*- How is the model trained, just using peak information, or the whole ChIA-PET data set?*

**Response:** More specifically, we get the peaks' coordinates of each interaction and calculate the input  features corresponding to each peak. And the original ChIA-PET interaction strength is the desired  output for the regression model. We divide the interactions of the cell line used in training into 75%  (training set) and 25% (testing set) and calculate the model's performance on the testing set. In addition,  we test the model on the data of the other three cell lines. For instance, if we train the model using  GM12878 data, we test the model on the 25% portion of the GM12878 data (testing set) and the data of  the three cell lines K562, H1, and HepG2.

*- One of the input data sets is CTCF motif orientation. How is this obtained - do they search for motifs  only within the Rad21 ChIP peaks, is CTCF ChIP data used? How do they deal with multiple motifs  appearing within a peak etc.? Is information on how well a given motif matches the consensus used?*

**Response:** Thank you for the comment. We apologize for not making this clear enough in the paper. We  search for motifs within the RAD21 peaks of the interactions (obtained as described in answering the  previous two comments). The CTCF motif orientation in these peaks’ locations can be obtained using  GimmeMotifs (van Heeringen and Veenstra 2011) as described in the “Getting the GC content and CTCF  motif orientation within peaks” subsection in the “Methods” section. GimmeMotifs reports only one  motif occurrence per peak (the highest-scoring motif). We did not use CTCF ChIP-seq data in this step.

The step of obtaining CTCF motif orientation can also be obtained in a more straightforward way as in  the source code available (please see https://git.biohpc.swmed.edu/s206442/chipr_nar/- /blob/main/Get_CTCF_orientation_flag.R). More specifically, we can use the locations and orientations of CTCF motifs available in the [http://compbio.mit.edu/encode-motifs/] (Kheradpour and Kellis 2014).  We did not use CTCF ChIP-seq data in this step. We checked the case when several motifs appeared in  the same peak. We found that this case happens in a small percentage of the peaks. And in most cases,  if several motifs are in the same peak, they have the same orientation. In a very small percentage (<2%  of interactions), the motifs in the same peak have different orientations. In this case, we focused only  about the orientation of the highest scoring motif.

*- Other input data include histone ChIP-seq and Rad21 ChIP-seq; how are these data sets used in the*

*model? Are peaks called, and peak positions and p-values used, or is a 'fold enrichment over input' track  generated?*

**Response:** Thank you for the comment. As described in addressing the previous comments, we obtain the peaks of interactions from the original ChIA-PET intrachromosomal interactions file. For these  available peak positions, we calculate the RPKM normalized read counts for different ChIP-seq inputs (as  described in subsection “ChIP-Seq data normalization” in the “Material and Methods” section). First, we  download the bigwig file of the ChIP-seq sample. We then convert the bigwig file to wig format, from  which we obtain the read counts and normalize them using the RPKM method. We made the code  needed for this process available [https://git.biohpc.swmed.edu/s206442/chipr/- /blob/main/prepare_chipseq_inputs.R].

*- In this section, to evaluate performance, the authors generated random values for the interaction  strengths to use as a control. This was a bit unclear. Particularly, do they correct for the genomic  distance between the peaks (they do this in some of the metrics used later)? How they calculate the  correlation was also not clear. Is the data set the interaction strength for all possible pairs of peaks, and  they find the correlation between prediction and data?*

**Response:** Thank you for the comment. In generating the control interaction strength, we have the  anchor peaks of the interactions from the original intrachromosomal ChIA-PET file and the  corresponding random value of interaction strength drawn from a negative binomial distribution having  the same mean and variance as the original interactions in the original ChIA-PET file.

We predict the interaction strength for the interactions in the original ChIA-PET intrachromosomal  interactions file. Thus, for each interaction, we have the original observed interaction strength and the  interaction strength value predicted by ChIPr. We calculated the correlation between these original  values and predicted values.

*- It would be useful to also offer some other metrics of the quality of prediction. Fraction of correctly or  incorrectly predicted interactions etc.*

**Response:** Thank you very much for pointing this out. We calculated the number of cell-type specific  interactions captured as strong interactions (strength >= 3) in the predictions of ChIPr. We got an  average accuracy of 74%, 78.5%, and 72.15% in the four cell lines using DNN-ChIPr, RF-ChIPr, and GB ChIPr, respectively (Fig. 4B-G in the manuscript). In addition, we introduced an equal number of random  interactions as a negative control. Those random interactions are expected to be weak. More than 95%  of these random interactions are accurately predicted as weak interactions by the three variants of  ChIPr. This experiment is described in the Section “ChIPr captures ChIA-PET identified cell-type dependent interactions” in the manuscript.

*- There are several statements in the text where a numerical value of p-value would help. e.g. "the  predicted outputs of the three different variants of ChIPr correlated significantly better with the..." could  quote some numbers here. Later they say results for different cell lines as "quite similar", again some  quantification would be useful.*

**Response:** Thank you for the comment. The correlation values are shown in the figures, and also, all the  p-values are mentioned in the figure legends. Moreover, about saying “quite similar,” we also depend  on the numerical values that are shown in the figures.

*4. Results: ChIA-PET at 25 and 5kbp bins*

*- Between this and the previous section, it is not clear what output the authors model actually gives. Is it  a binned map from which they then call peaks, or is it a set of peaks from which they then generate a  binned map. Or are these two separately trained models?*

**Response:** Thank you for the comment. The model gives interaction strength between peaks. The peak  length is in the range of 2 Kb. It may be slightly smaller or bigger than that. From interactions between  peaks, we build bin-based contact maps. We do that by summing all interactions whose anchor peaks lie  within the same bin. For instance, to build a 25 Kb bins contact map, we have a square matrix where  each entry represents the interaction strength between two 25 Kb bins. To get the value of interaction  strength between a pair of bins, we sum all the interactions whose anchor peaks fall within these two  bins. To clarify this part in the manuscript, we added a new subsection “Constructing contact maps from  peak-level interactions” in the “Material and Methods” section.

*- In this section they use a weighted sum of Pearson correlation coefficients from different distance  strata as a quality metric. More detail of this calculation would be useful. For example, a 'smoothing half  size' is mentioned, but not explained in the main text. A diagram of some sort might help.*

**Response:** Thank you for the comment. We use stratum-adjusted correlation coefficient (SCC) (Yang,  Zhang et al. 2017) to measure the similarity between contact maps. SCC is described in  [https://github.com/TaoYang-dev/hicrep].

*- There is a statement that predicted maps correlate significantly with the original maps. Again, some  numbers in the text would be helpful.*

**Response:** Thank you for pointing this out. We added the p-values to the legend of Fig. 3 in the  manuscript.

*5. Results: ChIPr captures both cell-type dependent and universal...*

*- The authors state that using all three ChIPr models would give the most robust view. This is not well  explained - should results from the 3 models be combined in some way, or just considered side by side?*

**Response:** Thank you very much for this comment. We recommend that the three models be used side  by side to confirm whether the interaction strength prediction is strong or weak. The main purpose of  ChIPr is to estimate cohesin-mediated interaction strength between anchor peaks when ChIA-PET data is  unavailable. If all three regression methods of ChIPr predict strong interaction (interaction strength >= 3)  between two anchor peaks, we expect that the interaction between these two peaks is truly strong.  Similarly, if the three of them predict weak interactions (interaction strength < 3), we expect that the  interaction is truly weak. However, in some cases, the three methods do not agree in predicting certain  interactions. In these cases, we consider the interaction weak or will not fully trust the predictions.

*6. Results: Contributions of input features to the ChIPr predictions*

*- The authors talk about a 'mean absolute error value', but don't explain what this means.*

**Response:** Thank you for the comment. It is the absolute value of the difference between the original  value and the predicted one,

mae = abs(y_true - y_pred)

*- Again in this section some numbers in the text could be useful. The authors talk about 'a small  difference' between cases where they do not include one of the ChIP-seq data sets and a 'bigger drop'  when they do not include two data sets.*

**Response:** Thank you for the comment. The numeric values are shown in Fig 7A, C, and D of the  manuscript.

*7. Results: minimal model section and CTCF section*

*- It is unclear whether authors are considering peak-level or binned interaction map level data here (or  both)*

**Response:** Thank you for your comment. ChIPr only detects interaction strength between peaks. Here  we consider peak-level interactions.

*- Again, how the authors obtain CTCF motif information is unclear. Do they search the entire genome for  CTCF motifs, or only Rad21 peaks? When they identify CTCF motifs, to they consider a score of how  closely the motif matches the consensus motif?*

**Response:** We search for motifs within the RAD21 peaks of the interactions (obtained as described in  answering the previous two comments). The CTCF motif orientation in these peaks’ locations can be  obtained using GimmeMotifs (van Heeringen and Veenstra 2011) as described in the “Getting the GC

content and CTCF motif orientation within peaks” subsection in the “Methods” section. GimmeMotifs  reports only one motif occurrence per peak (the highest-scoring motif). We did not use CTCF ChIP-seq  data in this step.

The step of obtaining CTCF motif orientation can also be obtained in a more straightforward way as  in the source code available (please see https://git.biohpc.swmed.edu/s206442/chipr/- /blob/main/Get_CTCF_orientation_flag.R). More specifically, we can use the locations and orientations  of CTCF motifs available in the [http://compbio.mit.edu/encode-motifs/] (Kheradpour and Kellis 2014).  We did not use CTCF ChIP-seq data in this step. We checked the case when several motifs appeared in  the same peak. We found that this case happens in a small percentage of the peaks. And in most cases,  if several motifs are in the same peak, they have the same orientation. In a very small percentage (<2%  of interactions), the motifs in the same peak have different orientations.

**References**

Bishop, C. M. and N. M. Nasrabadi (2006). Pattern recognition and machine learning, Springer. Breiman, L. (2001). "Random forests." Machine learning **45**(1): 5-32.

Grubert, F., et al. (2020). "Landscape of cohesin-mediated chromatin loops in the human  genome." Nature **583**(7818): 737-743.

Kheradpour, P. and M. Kellis (2014). "Systematic discovery and characterization of regulatory  motifs in ENCODE TF binding experiments." Nucleic acids research **42**(5): 2976-2987.

Ramanand, S. G., et al. (2020). "The landscape of RNA polymerase II–associated chromatin  interactions in prostate cancer." The Journal of clinical investigation **130**(8).

van Heeringen, S. J. and G. J. C. Veenstra (2011). "GimmeMotifs: a de novo motif prediction  pipeline for ChIP-sequencing experiments." Bioinformatics **27**(2): 270-271.

Wade, C. (2020). Hands-On Gradient Boosting with XGBoost and scikit-learn: Perform accessible  machine learning and extreme gradient boosting with Python, Packt Publishing Ltd.

Yang, T., et al. (2017). "HiCRep: assessing the reproducibility of Hi-C data using a stratum adjusted correlation coefficient." Genome research **27**(11): 1939-1949.

**2nd round**

**Reviewer 2**

I have read the authors' revised manuscript, and have found it to be much improved. Particularly, they have added a lot of detail to the methods section. I think that most of the detailed points that I raised have now been addressed. They have now also made their code available (though I was not able to get this to run).

However, I still think that this study is not of broad enough interest or high enough significance for the readership of Genome Biology. There have been many other recent paper using similar methods (for a review see, e.g., PMID:33454752 or PMID:35832620), and I think the present work makes an incremental contribution rather than a significant advance. I think it would be more appropriate in a more focused Bioinformatics journal.

**Author’s response**

Reviewer #2: I have read the authors' revised manuscript, and have found it to be much improved. Particularly, they have added a lot of detail to the methods section. I think that most of the detailed points that I raised have now been addressed. They have now also made their code available (though I was not able to get this to run).

**Response:** We are glad we were able to make the paper much better and address most of the detailed points the reviewer raised.

However, I still think that this study is not of broad enough interest or high enough significance for the readership of Genome Biology. There have been many other recent paper using similar methods (for a review see, e.g., PMID:33454752 or PMID:35832620), and I think the present work makes an incremental contribution rather than a significant advance. I think it would be more appropriate in a more focused Bioinformatics journal.

**Response:** We thank the reviewer for his comment. Our study originally focused on the prediction of RAD21-mediated interactions because, despite of the importance of this cohesion-subunit in shaping the 3D genome ^1^, none of the computational chromatin interaction prediction methods focuses specifically on its interactions prediction.

Since there are no other tools for predicting cohesin-mediated interactions’ strength, we compared ChIPr to another tool originally used to predict Hi-C interactions. 3Dpredictor can predict Hi-C contact maps at relatively high resolutions like 5 and 25 Kb bins using distance, CTCF binding information, and RNA-seq data as inputs ^2^. To use 3Dpredictor to predict cohesin-mediated interactions, we constructed 25 Kb contact maps of the K562 cell line using its original RAD21 ChIA-PET interactions. We used the contact maps of odd chromosomes to train 3Dpredictor and predicted the contact maps of even chromosomes. We also trained the three variants of ChIPr (DNN-ChIPr, RF-ChIPr, and GB-ChIPr) using the K562 cell line odd chromosomes and predicted the interactions of even chromosomes at the peak level resolution using ChIPr. To compare the outputs of both methods, we constructed the contact maps of even chromosomes using the predictions of ChIPr at 25 Kb resolution. We found the three variants of ChIPr significantly outperform 3Dpredictor according to stratum adjusted correlation coefficient (SCC) (**Fig. 12A** in the revised manuscript).

Also, we trained ChIPr with K562 Hi-C data Chr1 at 5Kb resolution. We predicted the Hi-C interactions of the other 22 chromosomes at 5Kb resolution, and we generated 25Kb resolution contact maps with these predictions. We also trained 3Dpredictor with K562 Hi-C data of Chr1 at 25 Kb resolution and predicted the contact maps of the other 22 chromosomes. We chose the K562 cell line because its training data for 3Dpredictor is available on their GitHub page [https://github.com/labdevgen/3Dpredictor/tree/master]. We compared the contact maps generated by both ChIPr and 3Dpredictor to the original contact maps of the K562 cell line. We found that ChIPr significantly outperforms 3Dpredictor for the three variants of ChIPr according to SCC (**Fig. 12B** in the revised manuscript). We also found the predicted Hi-C maps using ChIPr are visually similar to the original Hi-C maps (**Fig. 12C and D** in the revised manuscript). This further indicates the outperformance of ChIPr over 3Dpredictor. It also shows, to our surprise, that ChIPr is valid to predict Hi-C data in addition to cohesin-mediated interactions’ strengths.

In addition, several methods were developed recently to predict Hi-C-like contact maps at high resolution for sub-megabase genomic distances like Orca ^3^ and C.Origami ^4^. While Orca predicts chromatin interactions based on genomic distance and sequence only, C.Origami also uses two cell-type-specific features: CTCF ChIP-seq data and ATAC-seq data. To compare ChIPr with C.Origami and Orca, we used the data they used to measure their performances. More specifically, we trained the three variants of ChIPr using IMR90 cell line Hi-C data using Chr1 only for training. We used the trained models to predict Hi-C interactions of the two cell lines, K562 and GM12878. We found that the predictions correlate with the original interactions of the two cell lines significantly better than control interactions (**Fig. 12E** in the revised manuscript). Then, for randomly selected 2Mb regions (see **Supp. Table 1** in the revised manuscript), we predicted 8 Kb resolution contact maps for the GM12878 cell line using models trained on K562 and IMR90 cell lines data, respectively. We also used C.Origami to predict the GM12878 contact maps of the same regions using a model trained on IMR90 cell line data. For comparison, we calculated the logged Hi-C intensity with iterative correction and eigenvector decomposition (ICE): (log(ICE normalized counts + 1)) for the contact maps predicted using ChIPr and the original Hi-C contact maps for the selected regions as suggested by C.Origami ^4^. We found that the predictions of the three variants of ChIPr using the two models trained on K562 and IMR90 cell lines data, respectively, correlate significantly higher with the original contact maps than the predictions of C.Origami (**Fig. 12F** in the revised manuscript). These results show that ChIPr can be used to predict Hi-C data efficiently and can outperform C.Origami.

In addition, we compared ChIPr with Orca using the data they used to measure their performance. We trained ChIPr on the Micro-C data of the H1  cell line ^5^. We used only Chr1 for training, and we tested the performance of ChIPr in predicting the other 22 chromosomes of the same cell line. We found that the predictions of the three variants of ChIPr correlate significantly better with the original interactions than control interactions (**Fig. 12G** in the revised manuscript). We also constructed 1 Mb contact maps at 5 Kb resolution for several regions in Chrs 8, 9, and 10 (the chromosomes used for validation and testing of the Orca-trained model). We predicted the contact maps of the same regions (see **Supp. Table 1** in the revised manuscript) using Orca [https://orca.zhoulab.io/] at 4 Kb resolution. For comparison, we performed the same pre-processing step suggested by Orca on ChIPr predictions and the corresponding original contact maps, iterative correction matrix balancing algorithm and adaptive coarse graining ^3^. Although the performance of both methods was very high, probably because we are testing using hold-out chromosomes of the same cell line used for training, we found that Pearson correlation values of the original maps with ChIPr predictions are significantly higher than those with Orca predictions (**Fig. 12H** in the revised manuscript). All these results show the outperformance of ChIPr over other 3D chromatin prediction methods. These results also indicate that ChIPr can efficiently predict high-resolution Micro-C contact maps (5 Kb resolution).

In conclusion, although ChIPr was originally developed to predict the interactions’ strengths of RAD21-mediated interactions, we found that ChIPr is valid for predicting Hi-C and Micro-C interactions efficiently. It also can outperform other recent chromatin interaction prediction methods. We believe that this makes ChIPr of particular interest to the scientific community. In the revised manuscript, we have added a new subsection in the “Results” section entitled “ChIPr outperforms other 3D chromatin interaction prediction methods” to show the comparison results mentioned above. In addition, we have added a new subsection in the “Methods” section entitled “Generating control interactions for Hi-C and Micro-C data” to describe how we obtained control interactions in the experiments described above.

1	Sun, Y. *et al.* RAD21 is the core subunit of the cohesin complex involved in directing genome organization. *Genome Biology* **24**, 1-27 (2023).

2	Belokopytova, P. S., Nuriddinov, M. A., Mozheiko, E. A., Fishman, D. & Fishman, V. Quantitative prediction of enhancer–promoter interactions. *Genome research* **30**, 72-84 (2020).

3	Zhou, J. Sequence-based modeling of three-dimensional genome architecture from kilobase to chromosome scale. *Nature genetics* **54**, 725-734 (2022).

4	Tan, J. *et al.* Cell-type-specific prediction of 3D chromatin organization enables high-throughput in silico genetic screening. *Nature biotechnology*, 1-11 (2023).

5	Krietenstein, N. *et al.* Ultrastructural details of mammalian chromosome architecture. *Molecular cell* **78**, 554-565. e557 (2020).

**3^rd^ round**

**Reviewer 2**

In their second revision, the authors have added additional analysis to their manuscript showing that their ChIPr algorithm can be used to predict HiC and Mirco-C interaction data, in addition to the cohesin ChIA-PET maps for which it was originally designed. Given that they have now compared the performance of their algorithm with other tools, I do think this may be of interest to some researchers within the community. I do believe that the manuscript should be published. However I still have reservations as to whether these results will be of high enough interest to the specific readership of Genome Biology (particularly as significant bioinformatics/programming expertise would be required to make use of the author's scripts, i.e., they are not presenting a software tool which could be broadly used by non-experts); the scope of the journal is though a matter for the editor.
